# SMAD4 TGF-**β**–independent function preconditions naive CD8^+^ T cells to prevent severe chronic intestinal inflammation

**DOI:** 10.1172/JCI151020

**Published:** 2022-04-15

**Authors:** Ramdane Igalouzene, Hector Hernandez-Vargas, Nicolas Benech, Alexandre Guyennon, David Bauché, Célia Barrachina, Emeric Dubois, Julien C. Marie, Saïdi M’Homa Soudja

**Affiliations:** 1 Tumor Escape Resistance and Immunity Department, Cancer Research Center of Lyon (CRCL), INSERM U1052, CNRS UMR 5286, Centre Léon Bérard (CLB) and University of Lyon 1, Lyon, France.; 2 Montpellier GenomiX, University of Montpellier, CNRS, INSERM, Montpellier, France.

**Keywords:** Gastroenterology, Immunology, Cellular immune response, Inflammatory bowel disease, T cells

## Abstract

SMAD4, a mediator of TGF-**β** signaling, plays an important role in T cells to prevent inflammatory bowel disease (IBD). However, the precise mechanisms underlying this control remain elusive. Using both genetic and epigenetic approaches, we revealed an unexpected mechanism by which SMAD4 prevents naive CD8^+^ T cells from becoming pathogenic for the gut. Prior to the engagement of the TGF-**β** receptor, SMAD4 restrains the epigenetic, transcriptional, and functional landscape of the TGF-**β** signature in naive CD8^+^ T cells. Mechanistically, prior to TGF-**β** signaling, SMAD4 binds to promoters and enhancers of several TGF-**β** target genes, and by regulating histone deacetylation, suppresses their expression. Consequently, regardless of a TGF-**β** signal, SMAD4 limits the expression of TGF-**β** negative feedback loop genes, such as *Smad7* and *Ski*, and likely conditions CD8^+^ T cells for the immunoregulatory effects of TGF-**β**. In addition, SMAD4 ablation conferred naive CD8^+^ T cells with both a superior survival capacity, by enhancing their response to IL-7, as well as an enhanced capacity to be retained within the intestinal epithelium, by promoting the expression of *Itgae*, which encodes the integrin CD103. Accumulation, epithelial retention, and escape from TGF-**β** control elicited chronic microbiota-driven CD8^+^ T cell activation in the gut. Hence, in a TGF-**β**–independent manner, SMAD4 imprints a program that preconditions naive CD8^+^ T cell fate, preventing IBD.

## Graphical abstract

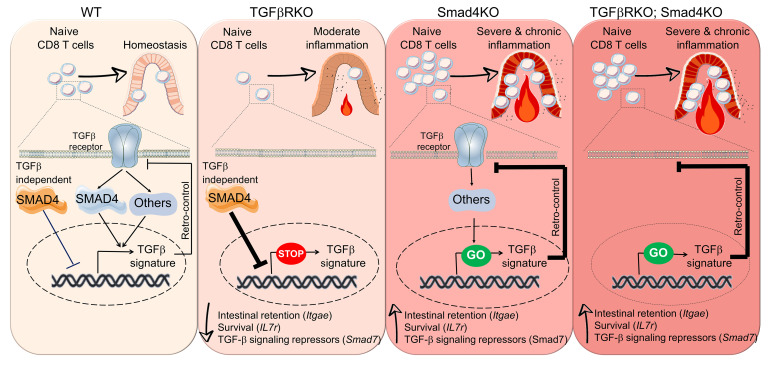

## Introduction

Inflammatory bowel disease (IBD) is a group of debilitating diseases that expose bearers to inflammation-associated cancers such as colorectal cancer (CRC) (1). An excessive immune reaction against microbiota is widely regarded as a common feature of IBD (2). Several immune-regulatory mechanisms prevent IBD, including the presence of the transforming growth factor β (TGF-β) cytokine. This cytokine is abundantly produced in the mammalian gut (3), and is implicated in T lymphocyte regulation by repressing numerous effector T cell functions and promoting regulatory T cell development, stability, and function (4). In line with the strong immune-regulatory effects of this cytokine, mice whose T cells do not respond to TGF-β succumb to severe and widespread autoimmunity shortly after birth (5, 6).

TGF-β binds to TGF-βRII and phosphorylates TGF-βRI. TGF-βRI then induces the phosphorylation of SMAD2 and SMAD3, which subsequently interact with either SMAD4 or tripartite motif–containing 33 (TRIM33). In addition, TGF-βR engagement triggers noncanonical signaling pathways, namely MAPK/MEK, JNK/p38, and AKT/PI3K (7). TGF-β signaling pathways regulate the expression of several genes, including TGF-β–repressor genes such as *Smad7* and *Ski*, thus generating a negative feedback loop to control TGF-β responses (8).

TGF-β signaling pathways work in concert, in opposition, or in competition, depending on the context. For instance, TRIM33 competes with SMAD4 to interact with SMAD2 and SMAD3, and in addition induces SMAD4 degradation (9, 10). Given this intricate interplay and interference, ablating one branch of TGF-β signaling may functionally impact the others positively or negatively, hampering their deciphering (11).

TGF-β signaling is altered in IBD and CRC patients (12). In particular, patients harboring SMAD4 germline mutations are predisposed to develop intestinal pathologies (13). The role of SMAD4 in intestinal homeostasis has been further reported in genetic mouse models, in which SMAD4 deficiency in T cells drove chronic inflammation and cancer (14, 15). Hence, given the complex interplay and interference between TGF-β pathways, the precise mechanisms governed by SMAD4 in T cells that prevent IBD remain to be deciphered.

Here, we show that contrary to an expected TGF-β–dependent function of SMAD4, SMAD4 in T cells protects mice from severe chronic intestinal inflammation in a TGF-β–independent manner. In addition, we show that CD8^+^ T cells play a critical role in this microbiota-driven immunopathology. Mechanistically, prior to TGF-β receptor engagement, SMAD4 acts at the chromatin level and imprints on naive CD8^+^ T cells a signature with effects opposite that of TGF-β, with direct impact on intestinal homeostasis. This imprinting at the naive stage preconditions CD8^+^ T cell functional and spatial commitment. Remarkably, inversely to TGF-β signaling, SMAD4 endows naive CD8^+^ T cells with an effector program but limits their accumulation and intestinal intraepithelial retention. Additionally, we uncover that, prior to TGF-β receptor engagement, SMAD4 inhibits the expression of TGF-β signaling repressors, such as *Smad7* and *Smurf2*, predisposing CD8^+^ T cells to an efficient TGF-β–mediated immunosuppression. Consequently, in the absence of SMAD4, CD8^+^ T cells massively colonize the intestinal epithelium and escape TGF-β–mediated immunosuppression, leading to their activation by the microbiota. Our findings assign an uncharacterized and crucial TGF-β–independent function to SMAD4 that programs naive CD8^+^ T cell fate, thus preventing severe chronic intestinal inflammation.

## Results

### SMAD4 in T cells has a TGF-β–independent function that protects mice from severe chronic intestinal inflammation.

**Figure 1:**
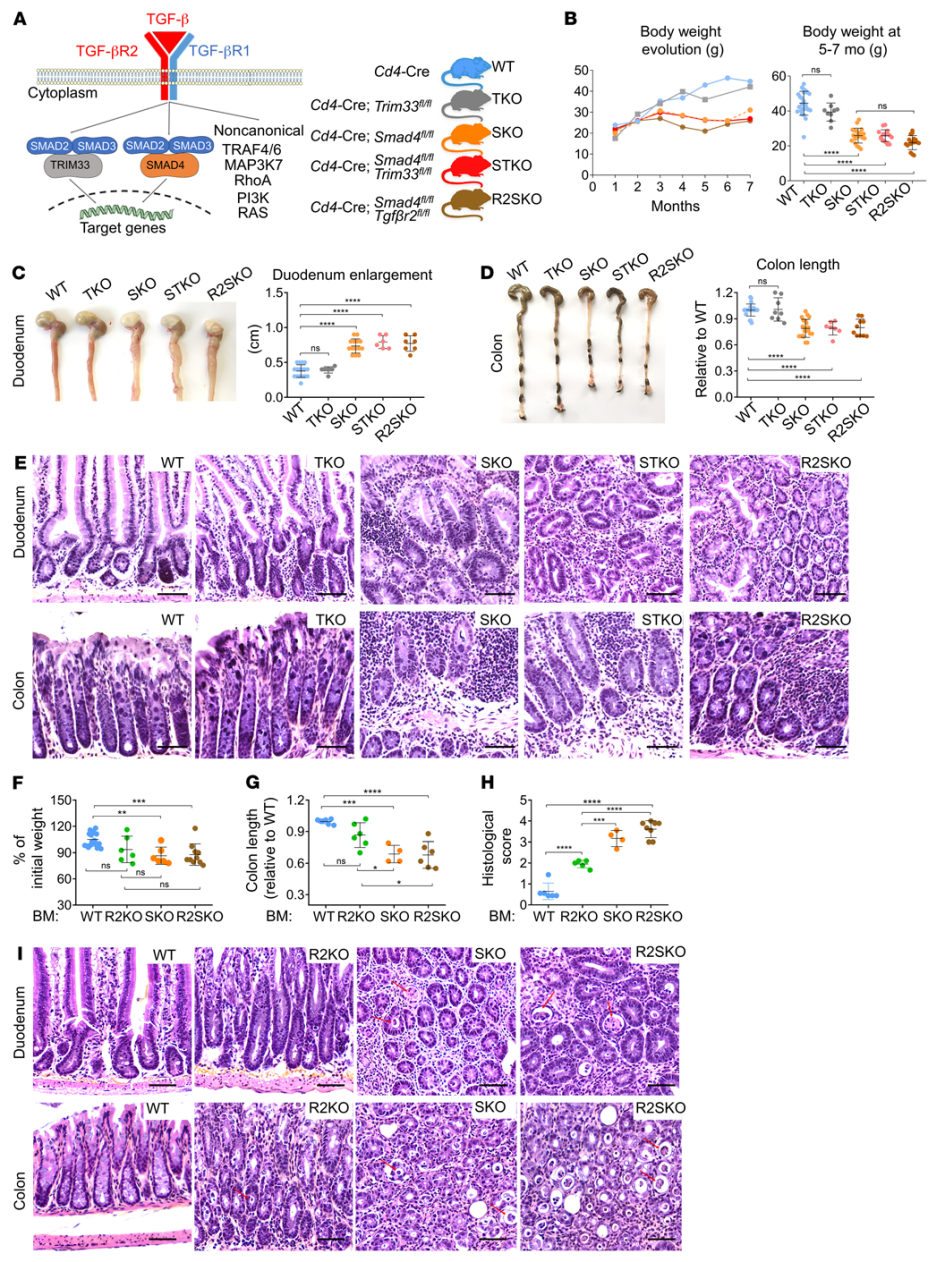
SMAD4 in T cells prevents severe chronic intestinal inflammation in a TGF-β–independent manner. (**A**) Scheme representing the different pathways of TGF-β signaling and the mouse models used. (**B**) On the left panel, body weight of mice from 1 to 7 months of age (*n* = 3 or more for each time point) and on the right panel, weight of the mice at 5 to 7 months of age (WT *n* = 27, TKO *n* = 9, SKO *n* = 21, STKO *n* = 15, and R2SKO *n* = 16). All mice were male. (**C** and **D**) Representative pictures of colon and duodenum, colon length, and duodenum enlargement of the different strains of mice at 7 months of age (*n* = 6–17). (**E**) Representative H&E staining of duodenum and colon sections at 7 months of age. Scale bars: 50 μm. Original magnification, ×20. (**F**–**I**) Irradiated RAG2-KO mice were reconstituted with WT, R2KO, SKO, or R2SKO BM cells. Percentage change in body weight between the beginning and the end of experiment (*n* = 6–19) (**F**), colon length (*n* = 4–18) (**G**), histological intestinal damage score (*n* = 4–8) (**H**), and representative H&E staining of duodenum and colon sections (**I**). Scale bars: 50 μm. Original magnification, ×20. Red arrows highlight crypt abscesses. All data represent at least 3 independent experiments (**C**–**I**) and are presented as mean ± SD. Each symbol represents an individual mouse (*n* = 4 or more for each group). Data were analyzed using 1-way ANOVA with Tukey’s test. **P < 0.01, ***P < 0.001, ****P < 0.0001. NS, not significant (*P* > 0.05).

Given the intricate interplay between TGF-β pathways, we first investigated the impact of other TGF-β signaling pathways in the gut inflammation described in mice lacking SMAD4 in T lymphocytes. To this end, we used the *CD4*-Cre conditional deletion system (16) to establish several mouse strains lacking one or several TGF-β signaling pathways in CD4^+^ and CD8^+^ T cells. We established mice with a single knockout of SMAD4 (SKO), single KO of TRIM33 (TKO), double KO of TRIM33 and SMAD4 (STKO), or double KO of TGF-βRII and SMAD4 (R2SKO) in T cells (Figure 1A and Supplemental Figure 1A; supplemental material available online with this article; https://doi.org/10.1172/JCI151020DS1). TKO, SKO, STKO, and R2SKO mice did not display any signs of autoimmunity even at an advanced age. However, strikingly, the weight of all mice lacking SMAD4 in T cells (SKO, STKO, and R2SKO) stopped increasing from 4 months of age onwards (Figure 1B). Postmortem analysis revealed an important intestinal inflammation in these animals, illustrated by an enlargement of the duodenum and a shortening of the colon (Figure 1, C and D). Histological analysis revealed massive immune cell infiltrations in the mucosa and submucosa in both the small intestine and the colon of these mice compared with WT and TKO mice. Additionally, evident hyperplasia, crypt abscesses, and strong intestinal crypt inflammation were detected in all mice lacking SMAD4 in T cells, indicative of a strong chronic inflammation in these animals (Figure 1E and Supplemental Figure 1B). Collectively, our data genetically demonstrate that the ablation of the remaining TGF-β pathways in SKO mice does not prevent mice from developing chronic intestinal inflammation.

Then, given that SMAD4 could mediate biological functions independently of TGF-β signaling in T cells (17, 18), we assessed whether a TGF-β–independent SMAD4 function in T cells could contribute to maintain intestinal homeostasis. TGF-βRII–deficient (R2KO) mice die within 3 to 4 weeks after birth (5, 6), while intestinal inflammation only develops at the adult age in SMAD4-deficient mice, so we employed a bone marrow–engrafted (BM-engrafted) mouse model to compare age-matched adult mice. We engrafted irradiated adult mice with BM from WT, R2KO, SKO, and R2SKO mice. Mice engrafted with R2KO, SKO, and R2SKO BM cells lost weight compared with WT BM–engrafted mice (Figure 1F). However, mice engrafted with BM cells from SKO and R2SKO mice developed more severe gut inflammation compared with those engrafted with R2KO BM cells, as evidenced by shorter colons, massive immune cell infiltrations, hyperplasia, and important mucosal damage (Figure 1, G–I, and Supplemental Figure 2A). These observations demonstrate that SMAD4 in T cells has a role that is TGF-β independent, protecting mice from severe chronic intestinal inflammation.

### CD8αβ T cells are a key effector population in intestinal immunopathologies observed in SMAD4-deficient mice.

**Figure 2:**
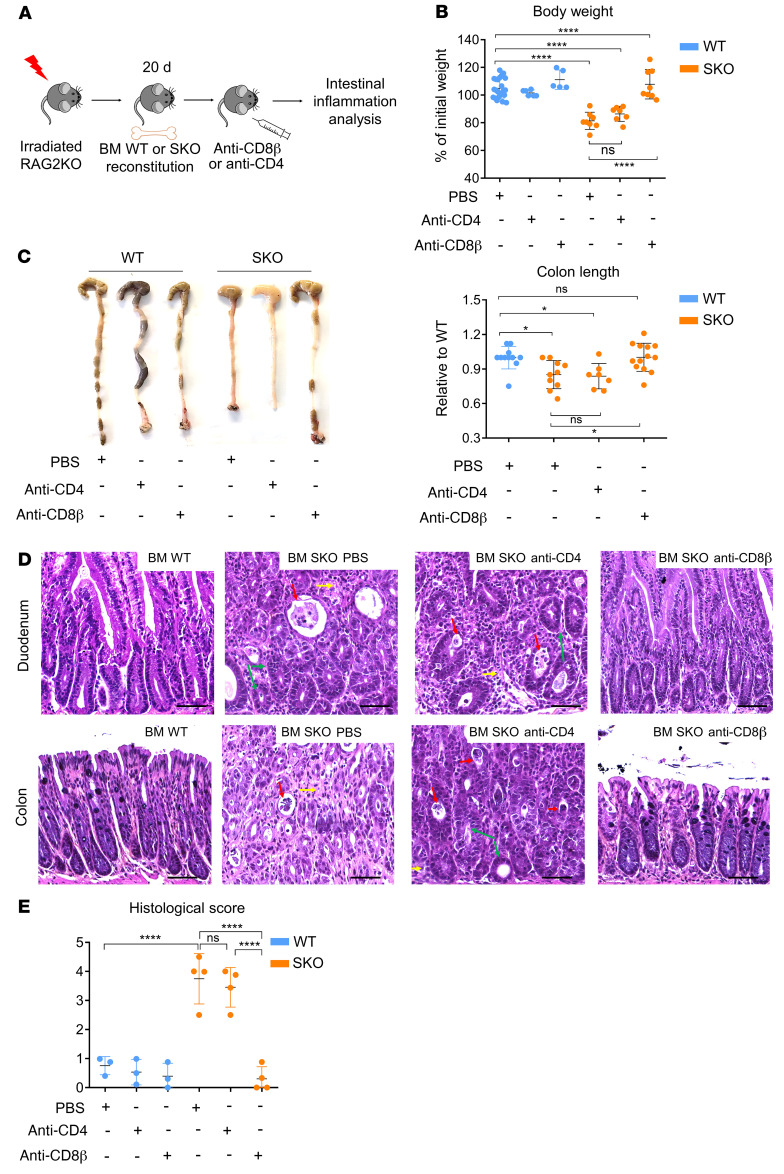
CD8αβ^+^ T cell depletion prevents intestinal inflammation upon SMAD4 deletion in T cells. (**A**) Scheme of the in vivo CD8β^+^ and CD4^+^ T cell depletion. (**B**) Body weight on days 35–40 after reconstitution with WT or SKO BM cells and treatment with anti-CD8β or anti-CD4 depleting antibody (*n* = 5–20). (**C**) Representative pictures of colons and colon length measurement of BM-reconstituted mice, treated with anti-CD8β or anti-CD4 depleting antibody (*n* = 7–14). (**D** and **E**) Representative H&E staining of duodenum and colon sections (**D**) and histological damage score (**E**) of irradiated mice reconstituted with WT or SKO BM cells and treated with anti-CD8β or anti-CD4 depleting antibody (*n* = 3–4). Scale bars: 50 μm. Original magnification, ×20. Red arrows highlight crypt abscesses: yellow arrows highlight immune infiltrate, green arrows highlight crypt irregularity. All data represent at least 3 independent experiments and are presented as mean ± SD. Each symbol represents an individual mouse and *n* = 3 or more for each group. Data were analyzed by unpaired 1-way ANOVA followed by Tukey’s test. *P < 0.05; ****P < 0.0001. NS, not significant (*P* > 0.05).

Since SMAD4 is deleted in both CD8^+^ and CD4^+^ T cells using the *CD4*-Cre conditional model (16), we next examined which effector T cell population mediates this intestinal immunopathology by using specific anti-CD4 and anti-CD8β depleting antibodies. To avoid undesirable long-term side effects of depleting antibody treatment, we used the BM-engrafted mouse models (Figure 2A). Flow cytometry analysis confirmed the effective ablation of conventional CD8αβ^+^ and CD4^+^ T cells in secondary lymphoid organs and in the gut without depleting the other populations, such as CD8αα^+^TCRαβ^+^ and CD8αα^+^TCRγδ^+^ populations (Supplemental Figure 3, A–C). Remarkably, SKO BM–engrafted mice treated with anti-CD8β did not exhibit weight loss and colon length reduction, in sharp contrast to anti-CD4– or isotype-control–treated mice (Figure 2, B and C, and Supplemental Figure 4A). Furthermore, histological examination showed a substantial decrease in immune cell infiltration and absence of hyperplasia and crypt abscesses in SKO BM–engrafted mice treated with anti-CD8β (Figure 2, D and E, and Supplemental Figure 4B). To ensure that CD8αβ T cells are also the main effectors driving intestinal damage in R2SKO-reconstituted mice, we conducted CD8^+^ T cell depletion in R2SKO BM–engrafted mice. Similarly, anti-CD8β treatment rescued R2SKO BM–engrafted mice from developing intestinal inflammation (Supplemental Figure 4C). Because a defect in T regulatory cells (Tregs) in SMAD4-deficient mice has been observed, in particular, in R2SKO mice (17), we further produced 1:1 mixed chimeras with WT and R2SKO BM cells to address whether the function of SMAD4 in CD8^+^ T cells is cell intrinsic. Mice transplanted with 1:1 WT and R2SKO BM cells kept losing weight and developed intestinal inflammation as judged by the shortness of the colon (Supplemental Figure 5, A–C), suggesting that the presence of WT Treg cells is not sufficient to prevent intestinal inflammation. Collectively, these results suggest an important role for CD8αβ^+^ T cells in contributing to the intestinal damage in SMAD4-deficient mice.

### SMAD4 prevents microbiota-driven accumulation and activation of CD8αβ^+^ T cells within the gut epithelium.

**Figure 3:**
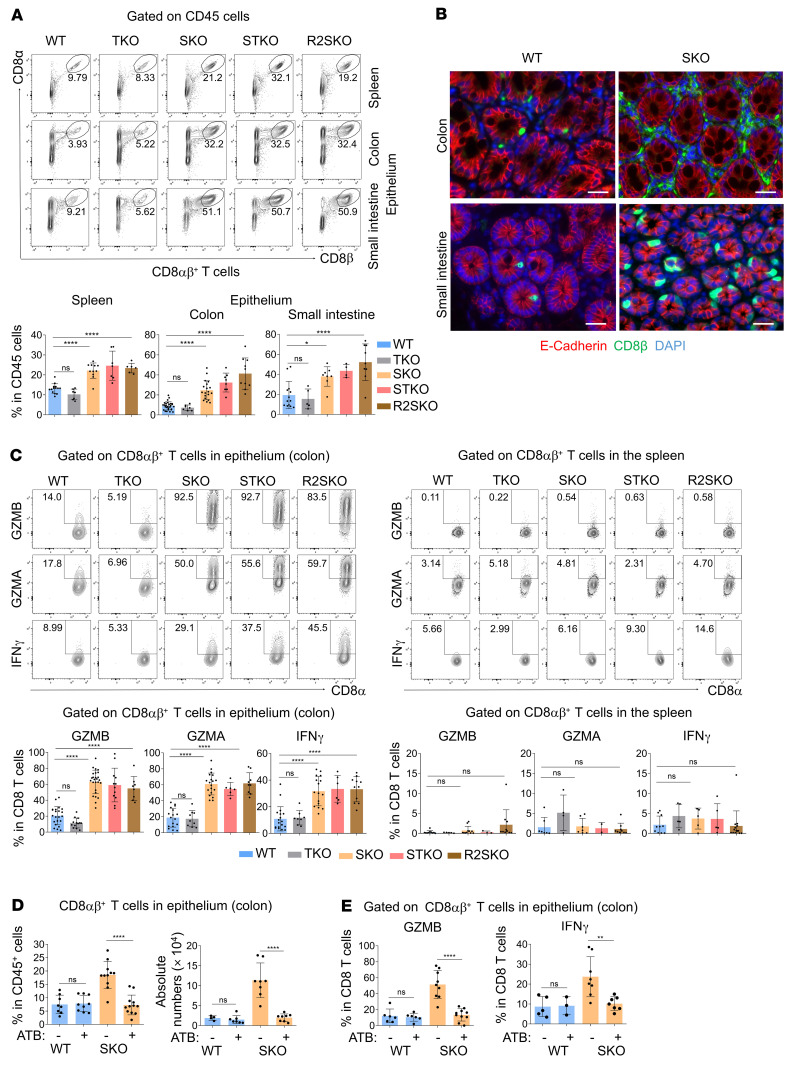
SMAD4 in T cells prevents microbiota-mediated accumulation and epithelial activation of CD8^+^ T cells. (**A**) Representative flow cytometry showing the frequency of CD8αβ^+^ T cells among CD45^+^ cells in the spleen and epithelium from the colon and small intestine of mice at 5–7 months of age (WT *n* = 14–29, TKO *n* = 5–7, SKO *n* = 8–19, STKO *n* = 4–8, and R2SKO *n* = 6–10). (**B**) Representative pictures showing immunofluorescence staining of CD8β (green), E-cadherin (red), and DAPI staining (blue) in the small intestine and colon sections of WT and SKO mice at 7 months of age. Scale bars: 50 μm. Original magnification, ×20. (**C**) Flow cytometry staining of GZMA, GZMB, and IFN-γ among splenic CD8αβ^+^ T cells and colonic CD8αβ^+^ intraepithelial lymphocytes (WT *n* = 8–21, TKO *n* = 4–13, SKO *n* = 5–26, STKO *n* = 2–12, and R2SKO *n* = 8–13). (**D** and **E**) Effect of antibiotic (ATB) treatment on the frequency, numbers, and activation of colonic intraepithelial CD8αβ^+^ T cells from WT and SKO mice at 5 months of age (WT without ATB *n* = 3–9, WT plus ATB *n* = 3–9, SKO without ATB *n* = 8–11, and SKO plus ATB *n* = 7–12). All data represent at least 3 independent experiments and are presented as mean ± SD. Each symbol represents an individual mouse. Data were analyzed by unpaired 1-way ANOVA with Tukey’s test. *P < 0.05, **P < 0.01, ****P < 0.0001. NS, not significant (*P* > 0.05).

We then assessed the mechanism by which SMAD4 in CD8αβ^+^ T cells prevents intestinal immunopathology. Strikingly, we observed in all SMAD4-deficient mice (SKO, STKO, and R2SKO) a substantial increase in the frequency and numbers of CD8αβ^+^ T cells in secondary lymphoid organs, as well as in the lungs, skin, colon, and small intestine, compared with WT or TKO mice (Figure 3, A and B, and Supplemental Figure 6, A and B). These data revealed a systemic accumulation of CD8αβ^+^ T cells in the absence of SMAD4. Besides this important accumulation, CD8αβ^+^ T cells from SKO, STKO, and R2SKO mice expressed large amounts of cytotoxic molecules, including granzymes A and B (GZMA and GZMB) or proinflammatory cytokines and chemokines such as IFN-γ, TNF-α, and CCL3 in the intestinal epithelium compared with WT and TKO mice (Figure 3C and Supplemental Figure 6, C and D). Importantly, the strong coexpression of the epithelial retention markers CD103 and GZMB suggests that the activated CD8αβ^+^ T cells were likely bona fide intraepithelial lymphocytes (IELs) (Supplemental Figure 6E). Remarkably, CD8αβ^+^ T cells from SMAD4-deficient mice were barely or not activated in the spleen, lung, skin, lymph nodes, and lamina propria of the intestine (Figure 3C and Supplemental Figure 6, F and G). This indicates that the activation of SMAD4-deficient CD8^+^ T cells within the intestinal epithelium is spatially restricted. It is noteworthy that CD4^+^ T cell frequency and number did not increase significantly in SKO mice (Supplemental Figure 7A). Moreover, the frequency of IL-17–producing cells in the gut was similar between SKO mice and age-matched WT mice (Supplemental Figure 7B). As further support for a potential role of CD8αβ^+^ T cells in human IBDs, we then utilized and analyzed publicly available sets of single-cell mRNA sequencing (scRNA-seq) data on CD8αβ^+^ T cells and CD4^+^ T cells from IBD patients (19, 20). Compared with different CD4^+^ T cell subsets, CD8αβ^+^ T cells produced more cytotoxic molecules (*GZMA*, *GZMB*, *GNLY*, and *PRF1*) as expected, confirming their cytotoxic feature. However, interestingly, CD8αβ^+^ T cells appeared also to produce higher levels of proinflammatory cytokines (*IFNG*), as well as chemokines (*CCL3*, *CCL4*, *CCL5*, and *XCL1*) in the gut mucosa of patients with IBD (Supplemental Figure 8, A and B), suggesting a potential key role for CD8αβ^+^ T cells in human pathogenesis of IBD.

Next, we investigated the mechanisms triggering intestinal epithelial activation of CD8αβ^+^ T cells in SMAD4-deficient mice. Given the importance of the microbiota in shaping intestinal immunity and promoting IBD (2), we hypothesized that commensal bacteria could be responsible for CD8αβ^+^ T cell–exacerbated intestinal epithelial activation. In order to confirm this scenario, SKO mice were treated with a cocktail of antibiotics previously established to profoundly reduce the amount of bacteria in the intestine (21). Strikingly, antibiotic treatment of SKO mice completely abrogated CD8αβ^+^ T cell accumulation in the gut epithelium (Figure 3D). In addition, the enhanced production of IFN-γ and granzymes in CD8αβ^+^ IELs was also abolished in antibiotic-treated SKO mice (Figure 3E). Hence, these data reveal that SMAD4 prevents the microbiota-driven activation of CD8αβ^+^ T cells within the epithelial layer of the intestine.

### The TGF-β–independent SMAD4 function restrains the TGF-β transcriptional signature and endows an effector predisposition in naive CD8^+^ T cells.

**Figure 4:**
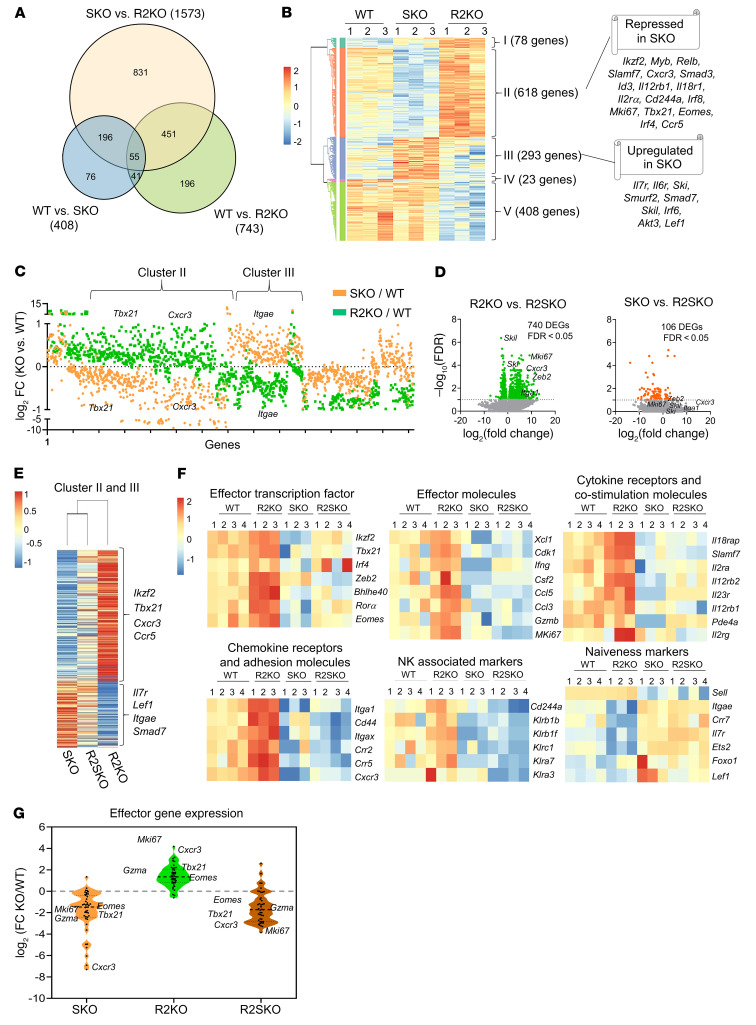
In the absence of TGF-βR signaling, SMAD4 restrains TGF-β signature in naive CD8^+^ T cells inversely of TGF-βR signaling. (**A**) Venn diagram showing the numbers of differentially expressed genes between WT (*n* = 3), R2KO (*n* = 3), and SKO (*n* = 3) naive F5 CD8αβ^+^ T cells after conducting whole-transcriptome sequencing. (**B**) Heatmap showing the hierarchical clustering of differentially expressed genes between WT, SKO, and R2KO F5 naive CD8αβ^+^ T cells. (**C**) Fold change (FC) (logarithmic scale) of gene expression of SKO over WT (in orange) and R2KO over WT (in green). DEGs correspond to those shown in heatmap in Figure 4B. (**D**) Volcano plot of RNA-seq data from R2KO (*n* = 3), SKO (*n* = 3), and R2SKO (*n* = 4) naive F5 CD8αβ^+^ T cells. The data for all genes are plotted as log_2_FC versus the –log_10_ of the adjusted *P* value. Genes selected as significantly different are highlighted as green and red dots. (**E**) Heatmap showing the log_2_FC expression of genes of cluster II and III highlighted in Figure 2B, and for each condition, the heatmap value corresponds to the KO relative to WT (average of 3 biological replicates). (**F**) Heatmaps showing the expression of genes linked to CD8^+^ T cell effector functions and genes linked to the naive and quiescent stage in WT, R2KO, SKO, or R2SKO F5 naive CD8αβ^+^ T cells. (**G**) Violin plot showing the relative expression of effector genes from R2KO (*n* = 3), SKO (*n* = 3), and R2SKO (*n* = 4) as compared to WT (*n* = 4) CD8^+^ T cells.

To go deeper into the molecular processes governing SMAD4-mediated control of CD8^+^ T cells, we next performed a global gene expression profile of CD8^+^ T cells from WT, SKO, and R2KO mice. In order to rule out any potential extrinsic effects of the inflammatory environment (IBD or autoimmunity) and focus precisely on the specific intrinsic role of SMAD4 in CD8^+^ T cells, we opted to use naive F5 TCR–transgenic CD8^+^ T cells, which recognize a peptide from the influenza virus (22). Conducting our investigation with a TCR transgenic model will allow us also to exclude the impact of the TCR repertoire in shaping the transcriptome of the T cells. To note, F5 TCR-transgenic mice in a RAG2-deficient background (RAG2-KO) do not develop any intestinal inflammation or autoimmunity (Supplemental Figure 9A). Unexpectedly, the comparison between SKO and R2KO naive F5 TCR CD8^+^ T cells resulted in a larger set of significantly differentially expressed genes (DEGs) (1573 genes) (FDR < 0.05) than the comparison between SKO and WT F5 TCR CD8^+^ T cells (408 DEGs) (Figure 4A), highlighting a wider molecular gap between SKO and R2KO naive CD8^+^ T cells. An unsupervised hierarchical clustering of all DEGs revealed 5 distinct clusters. Strikingly, DEGs in which SMAD4 deletion and TGF-βRII deletion show a distinct expression pattern (clusters II, III, and V) represent more than 92% of all DEGs, indicating a wide transcriptional disparity between SKO and R2KO CD8^+^ T cells (Figure 4B and Supplemental Figure 9B). More importantly, the large majority of the DEGs behaved oppositely between SKO and R2KO CD8^+^ T cells (clusters II and III) (Figure 4, B and C). Thus, this wide transcriptional disparity suggests that it is likely the result of an opposite effect between SMAD4 and TGF-β signaling. To assess whether this marked transcriptional opposition orchestrated by SMAD4 is not mediated by a TGF-β signal, we then conducted genome-wide RNA-seq in R2SKO F5 TCR CD8^+^ T cells. This analysis unveiled a larger set of DEGs (740 genes) in the comparison between R2SKO and R2KO CD8^+^ T cells, in contrast to the comparison between SKO and R2SKO CD8^+^ T cells (106 DEGs) (Figure 4D). Furthermore, in R2SKO CD8^+^ T cells, the absence of SMAD4 largely reversed the gene expression observed after total TGF-β signaling deletion (R2KO) (Figure 4E). Thus, in the absence of TGF-β, SMAD4 acts as a basal repressor of the TGF-β transcriptional landscape in CD8^+^ T cells.

Next, we determined functional outcomes of this transcriptional divergence. A deeper examination of the divergent DEGs highlights many genes belonging to the T cell effector program. In contrast to SKO and R2SKO, R2KO naive CD8^+^ T cells exhibited an enhanced expression of genes encoding effector molecules (e.g., *IFNG*, *CCL5*) and transcription factors known to direct T cell effector differentiation (e.g., *Tbx21*, *Zeb2*, *Irf4*). Conversely, genes associated with quiescence/naiveness (e.g., *Lef1*, *Itgae*, *IL7r*, *Ets2*) were repressed in R2KO but upregulated in SKO and R2SKO CD8^+^ T cells (Figure 4F). A gene set enrichment analysis (GSEA) of all DEGs and the expression of 43 selected genes associated with T cell activation indicated that SMAD4 deletion in R2SKO CD8^+^ T cells abrogates effector gene expression observed in the single TGF-βRII deletion (R2KO) (Figure 4G and Supplemental Figure 9, C and D). Overall, our data reveal that before TGF-βR engagement, SMAD4 restricts transcriptional and functional TGF-β signatures in CD8^+^ T cells.

### SMAD4 preconditions naive CD8^+^ T cells to TGF-β–mediated immunosuppression.

**Figure 5:**
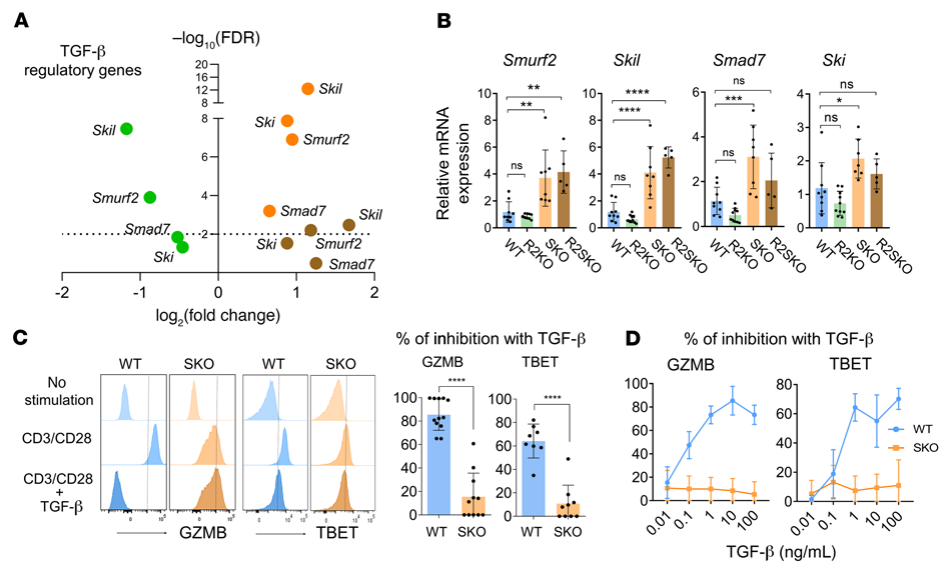
SMAD4 depletion promotes expression of TGF-β repressors and impedes TGF-β response in CD8^+^ T cells. (**A**) Volcano plot showing TGF-β inhibitory genes in SKO (*n* = 3, orange), R2KO (*n* = 3, green), and R2SKO (*n* = 4, brown) F5 naive CD8αβ^+^ T cells, all relative to WT (*n* = 4). (**B**) Quantitative RT-PCR analysis of the expression of indicated TGF-β regulatory genes in F5 naive CD8αβ^+^ T cells from spleen of WT, R2KO, SKO, and R2SKO mice. These mice are different from those used for RNA-seq data (*n* = 5–9). (**C** and **D**) Flow cytometry data showing inhibition of GZMB (*n* = 11–12 per group) and TBET (*n* = 8–9 per group) after anti-CD3/anti-CD28 stimulation of WT or SKO CD8αβ^+^ T cells with or without recombinant TGF-β at 10 ng/mL (**C**) or different concentrations (*n* = 5 mice per group) (**D**). The percentage of CD8αβ^+^ T cell inhibition was determined by calculating the ratio between anti-CD3/anti-CD28 plus TGF-β and anti-CD3/anti-CD28 alone. All data represent at least 2 independent experiments and are presented as mean ± SD. Each symbol represents an individual mouse. Data were analyzed by unpaired Student’s *t* test (**C**) and 1-way ANOVA with Tukey’s test for the other panels. *P < 0.05, **P < 0.01, ***P < 0.001, ****P < 0.0001. NS, not significant (*P* > 0.05).

Since SMAD4 deletion limits effector predisposition of naive CD8^+^ T cells, a compensatory mechanism must allow microbiota-driven activation of intestinal CD8^+^ T cells in SKO mice. Intriguingly, genes encoding potent TGF-β signaling repressors (e.g., *Smad7*, *Ski*, *Skil*, and *Smurf2*) were enhanced in SKO and R2SKO compared with R2KO CD8^+^ T cells (Figure 5A). We validated the overexpression of those genes by quantitative real-time PCR (RT-PCR) on naive F5 CD8^+^ T cells, as well as on polyclonal SKO and R2SKO CD8^+^ T cells but not in WT and TKO CD8^+^ T cells (Figure 5B and Supplemental Figure 10A), confirming that this overexpression was not restricted to a specific TCR repertoire. The expression defect of TGF-β repressors in R2KO CD8^+^ T cells confirms that they are TGF-β target genes (8). Since the double deletion of TGF-βRII and SMAD4 (R2SKO) restored the gene expression of TGF-β repressors (Figure 5B and Supplemental Figure 10A), this demonstrates that SMAD4 inhibits the expression of TGF-β repressors in a TGF-β–independent manner. Because expression of TGF-β repressors has been associated with a profound defect in T cell responses to TGF-β in IBD (12), we examined the effect of TGF-β on SMAD4-deficient CD8^+^ T cell activation. While TGF-β strongly inhibited GZMB and TBET expression in activated WT CD8^+^ T cells even at low doses, their expression was maintained even at high concentrations of TGF-β in SKO CD8^+^ T cells (Figure 5, C and D). Thus, these observations reveal that SMAD4 ablation limits the immune-regulatory effects of TGF-β on CD8^+^ T cells and demonstrates that SMAD4 is important for TGF-β–mediated immunosuppression.

### Overactivation of the remaining TGF-β signaling pathways in SMAD4-deficient mice does not prevent microbiota-induced CD8^+^ T cell activation in vivo and intestinal immunopathologies.

**Figure 6:**
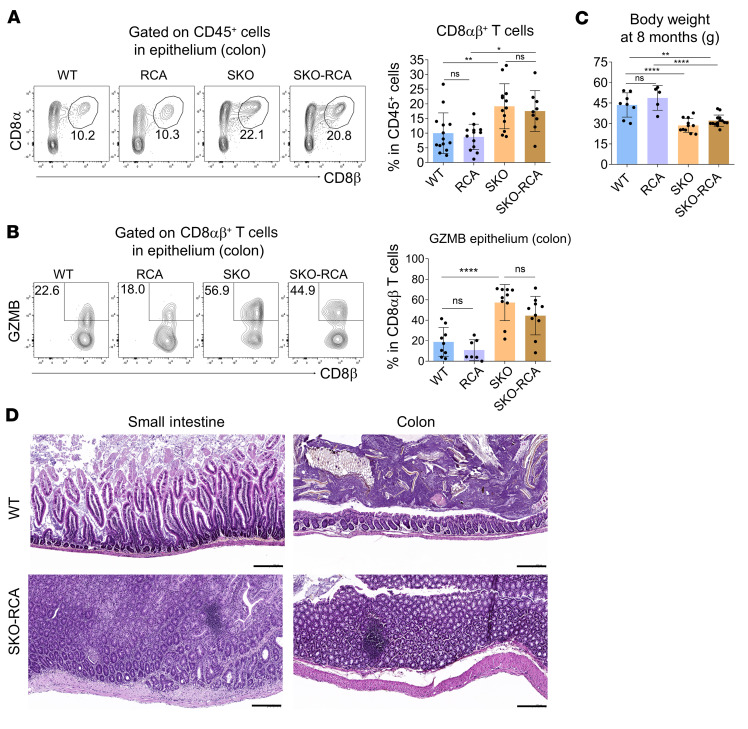
Overactivation of the remaining TGF-β signaling pathways in SMAD4-deficient mice does not rescue mice from intestinal immunopathologies. (**A** and **B**) Flow cytometry plots showing the frequency of CD8αβ^+^ T cells among CD45^+^ cells present within the colonic epithelium of WT (*n* = 14), RCA (*n* = 13), SKO (*n* = 13), and SKO-RCA (*n* = 9) mice (**A**), and intracellular staining for GZMB among colonic epithelial CD8αβ^+^ T cells (**B**). (**C**) The body weight in grams of WT (*n* = 8), RCA (*n* = 5), SKO (*n* = 11), and SKO-RCA (*n* = 14) male mice. (**D**) H&E staining of duodenum and colon sections from mice at 8 months of age. Scale bars: 200 μm. Original magnification, ×20. All data represent at least 3 independent experiments and are presented as mean ± SD. Each symbol represents an individual mouse and *n* = 5 or more for each group. Data were analyzed by 1-way ANOVA with Tukey’s test.*P < 0.05, **P < 0.01, ****P < 0.0001. NS, not significant (*P* > 0.05).

Because TGF-β is highly enriched in the gut (3) and represses T cell activation (4), this impaired response to TGF-β could contribute to chronic microbiota-driven CD8^+^ T cell activation. In order to confirm this assumption in vivo, we forced the activation of SMAD4-independent pathways of TGF-β signaling by crossing SKO mice with mice bearing a conditionally expressed, constitutively active form of TGF-βRI (RCA) (23). In the resulting SKO-RCA mice, CD8αβ^+^ T cells were as abundant and activated in the gut epithelium as in SKO mice (Figure 6, A and B), and more importantly, SKO-RCA mice developed IBD (Figure 6, C and D). Hence, remaining TGF-β signaling pathways are unable to compensate for SMAD4 loss in vivo. Collectively, these data suggest that the TGF-β–independent function of SMAD4 facilitates the response of CD8^+^ T cells to TGF-β by restraining the expression of TGF-β repressors in a feedforward mechanism (prior to any TGF-β signal), and SMAD4 appears to be a major mediator of immune-regulatory effects of TGF-β.

### TGF-β–independent function of SMAD4 restrains homeostatic survival of CD8^+^ T cells.

**Figure 7:**
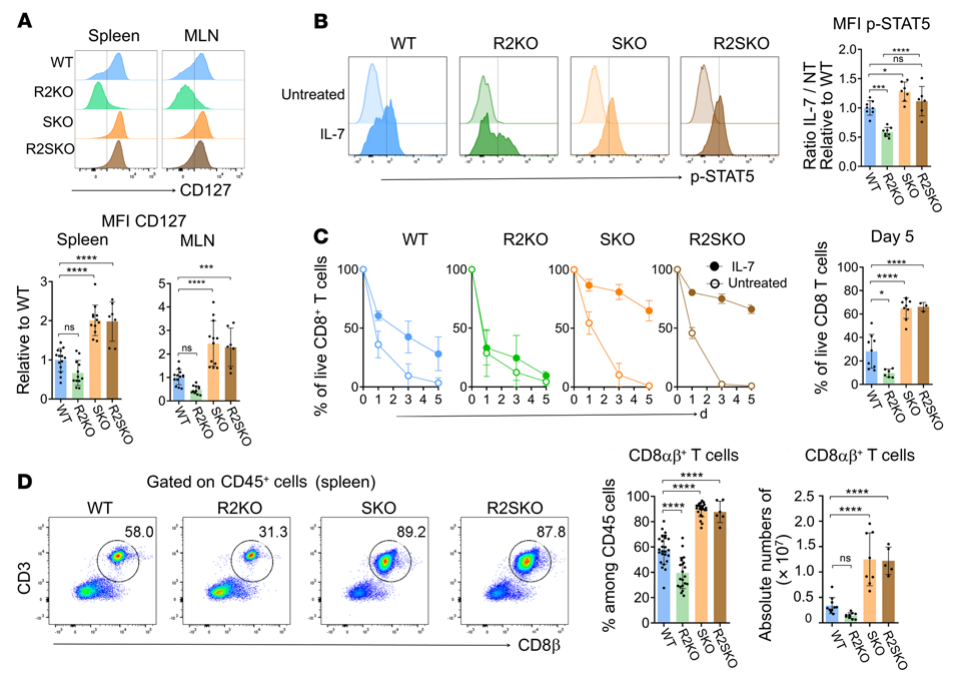
SMAD4 promotes homeostatic survival of CD8αβ^+^ T cells in an opposite way to TGF-βR signaling. (**A**) Flow cytometry staining of CD127 (IL-7Rα) on WT (*n* = 14), R2KO (*n* = 12), SKO (*n* = 12), and R2SKO (*n* = 7) F5 naive CD8αβ^+^ T cells from spleen and MLNs and histograms showing mean fluorescence intensity (MFI) of CD127 relative to WT. (**B**) Flow cytometry staining of phosphorylated STAT5 (p-STAT5) in WT (*n* = 8), R2KO (*n* = 8), SKO (*n* = 7), and R2SKO (*n* = 6) F5 naive CD8αβ^+^ T cells after in vitro IL-7 treatment and histograms showing relative MFI of p-STAT5 relative to WT. NT, not treated. (**C**) Survival monitoring of WT (*n* = 6), R2KO (*n* = 7), SKO (*n* = 6), and R2SKO (*n* = 3) naive F5 CD8αβ^+^ T cells treated or not with IL-7. (**D**) Flow cytometry data showing the frequency with absolute numbers of F5 naive CD8αβ^+^ T cells among CD45^+^ cells in the spleen of 3-month-old WT (*n* = 9), R2KO (*n* = 6), SKO (*n* = 9), and R2SKO (*n* = 3) F5 mice. All data represent at least 3 independent experiments and are presented as mean ± SD. Each symbol represents an individual mouse. Data were analyzed by 1-way ANOVA with Tukey’s test. *P < 0.05, ***P < 0.001, ****P < 0.0001. NS, not significant (*P* > 0.05).

Given that R2KO mice, in which T cells do not respond to TGF-β signals, do not exhibit as severe gut inflammation as SKO and R2SKO mice (Figure 1, F–I), additional factors might enhance the intestinal inflammation in SKO and R2SKO mice. Strategically, we focused on genes crucial for CD8^+^ T cell homeostasis and epithelial layer retention that are similarly affected in SKO and R2SKO and inversely affected in R2KO CD8^+^ T cells. Our first target was IL-7R (also termed CD127) since it plays a crucial and nonredundant role in homeostatic survival of CD8^+^ T cells (24) and recent studies associated IL-7 signaling overactivation and IBD (25). In line with the RNA-seq data, flow cytometry analysis validated that naive F5 TCR SMAD4-deficient (SKO and R2SKO) CD8^+^ T cells overexpressed IL-7R compared with WT CD8^+^ T cells, in sharp contrast to R2KO CD8^+^ T cells (Figure 7A). Similarly, we observed this upregulation of IL-7R in naive as well as memory CD8^+^ T cells with a polyclonal TCR repertoire (Supplemental Figure 11A). Consistent with the level of IL-7R expression, STAT5 phosphorylation, which is induced upon IL-7 stimulation, was slightly enhanced in SKO and R2SKO CD8^+^ T cells, and impaired in R2KO CD8^+^ T cells (Figure 7B). A time-course analysis of survival demonstrated that, in sharp contrast to R2KO CD8^+^ T cells, IL-7 treatment impressively increases the survival of SKO and R2SKO CD8^+^ T cells compared with WT cells (Figure 7C). Accordingly, we observed a substantial increase in the absolute number and the proportion of CD8^+^ T cells in secondary lymphoid organs from SKO and R2SKO F5 transgenic mice, unlike R2KO mice (Figure 7D and R. Igalouzene, unpublished observations). These findings reveal a critical role for the TGF-β–independent SMAD4 function in restraining CD8^+^ T cell accumulation by repressing the IL-7 response.

### TGF-β–independent function of SMAD4 restrains gut epithelial retention of CD8^+^ T cells.

**Figure 8:**
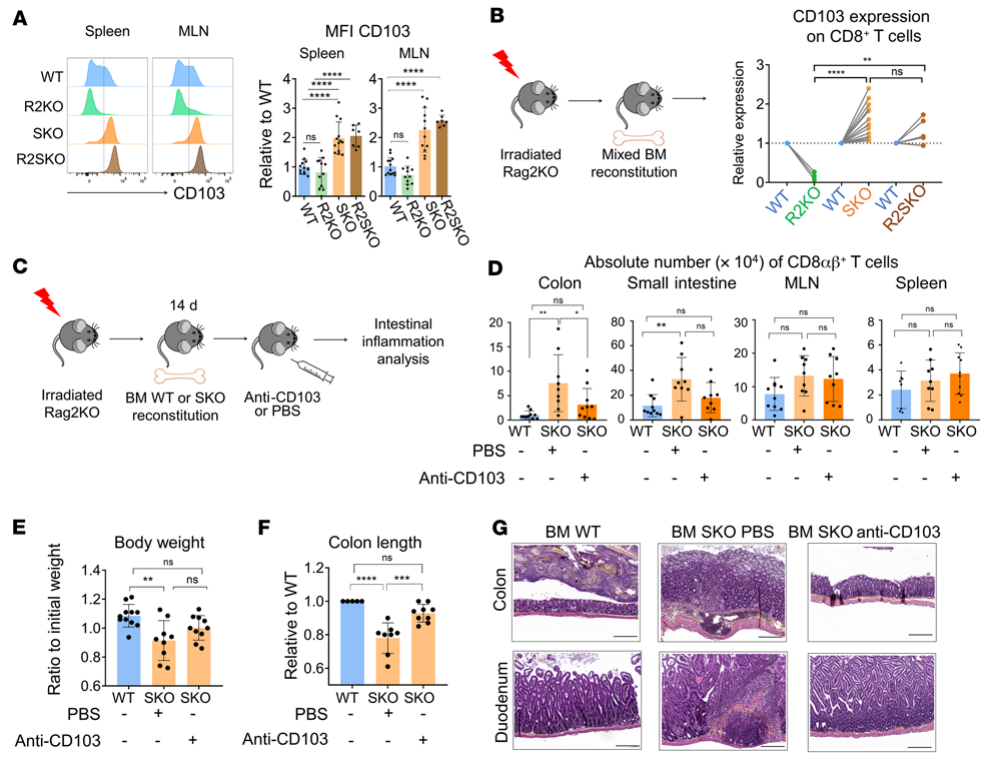
SMAD4 promotes gut epithelial retention of CD8αβ^+^ T cells in an opposite way to TGF-βR signaling. (**A**) Representative histograms of CD103 expression by F5 naive CD8αβ^+^ T cells — WT (*n* = 12), R2KO (*n* = 12), SKO (*n* = 13), and R2SKO (*n* = 7) — and histogram of the relative MFI of CD103 expression compared to WT. (**B**) Flow cytometry–assessed expression of CD103 on R2KO (*n* = 3), SKO (*n* = 14), and R2SKO (*n* = 6) CD8^+^ T cells from spleen, shown as relative to WT counterpart, after mixed BM experiment. (**C**–**G**) Experimental procedure for anti-CD103 blocking treatment (**C**), CD8^+^ T cell absolute number (**D**), body weight change from initial weight (**E**), colon length relative to WT (**F**), and H&E staining of duodenum and colon sections (**G**) of irradiated mice reconstituted with WT (*n* = 11) or SKO BM cells and treated (*n* = 9) or not (*n* = 9) with anti-CD103 blocking antibody. Scale bars: 200 μm. Original magnification, ×20. All data represent at least 3 independent experiments and are presented as mean ± SD. Each symbol represents an individual mouse. Data were analyzed by an unpaired Student’s *t* test (**F**) and 1-way ANOVA followed by Tukey’s test for other panels. *P < 0.05; **P < 0.01; ***P < 0.001; ****P < 0.0001. NS, not significant (*P* > 0.05).

In addition to *Il7r*, *Itgae* (which encodes CD103) was also aberrantly upregulated in CD8^+^ T cells from SKO mice. CD103 is of great interest, as it elicits T cell retention within the intestinal epithelial layer (26). In agreement with the RNA-seq data, SKO and R2SKO naive CD8^+^ T cells exhibited an enhanced level of CD103 (Figure 8A). Furthermore, CD103 expression was also markedly increased in polyclonal memory/experienced CD44^+^CD8^+^ T cells from SKO and R2SKO mice, but not those from WT or TKO mice (Supplemental Figure 12, A and B). It is noteworthy that SMAD4 ablation did not translate into an increase in CD103 expression in CD4^+^ T cells, in sharp contrast to CD8^+^ T cells (Supplemental Figure 12, C and D). We further generated 1:1 mixed chimeras with WT and R2KO, SKO, or R2SKO BM cells to address whether the expression of CD103 is intrinsic or induced by the inflammatory environment. Flow cytometry staining showed that the upregulation of CD103 in SKO and R2SKO CD8^+^ T cells is intrinsic because WT CD8^+^ T cells were not affected by this upregulation (Figure 8B). In correlation with the absence of CD103 expression, R2KO CD8^+^ T cells were less enriched in the intestinal epithelium compared with R2SKO and SKO CD8^+^ T cells (Supplemental Figure 12, E–G). This impaired epithelial tropism of R2KO CD8^+^ T cells may explain the milder intestinal inflammation observed in those mice compared with R2SKO mice. In line with this assumption, we next addressed whether the exacerbated expression of CD103 plays a role in the IBD observed in SKO mice. We treated BM-engrafted mice with a blocking antibody that specifically recognizes CD103 (Figure 8C). This CD103 blockade led to a decrease in CD8^+^ T cell numbers within the intestinal epithelium of SKO mice, without altering their accumulation in secondary lymphoid organs such as the spleen and mesenteric lymph nodes (MLNs) (Figure 8D). Although this treatment did not fully restore body weight in SKO reconstituted mice, the colon length and immunohistological analysis highlighted clear improvement (Figure 8, E–G). The colon length reduction and the mucosal damage due to immune infiltration were alleviated, indicating a beneficial effect of CD103 blockade in SKO mice. Globally, in addition to the impaired response to TGF-β–mediated immune-regulatory functions, SMAD4 disruption promotes IL-7 responsiveness and epithelial retention of CD8^+^ T cells in a TGF-β–independent manner. This combination of altered positioning and increased numbers of CD8^+^ T cells in the gut epithelium of SMAD4-deficient mice leads to severe chronic intestinal inflammation compared with R2KO mice.

### TGF-β–independent function of SMAD4 that restrains CD103 and IL-7R occurs in CD8^+^ T cells at the naive stage.

**Figure 9:**
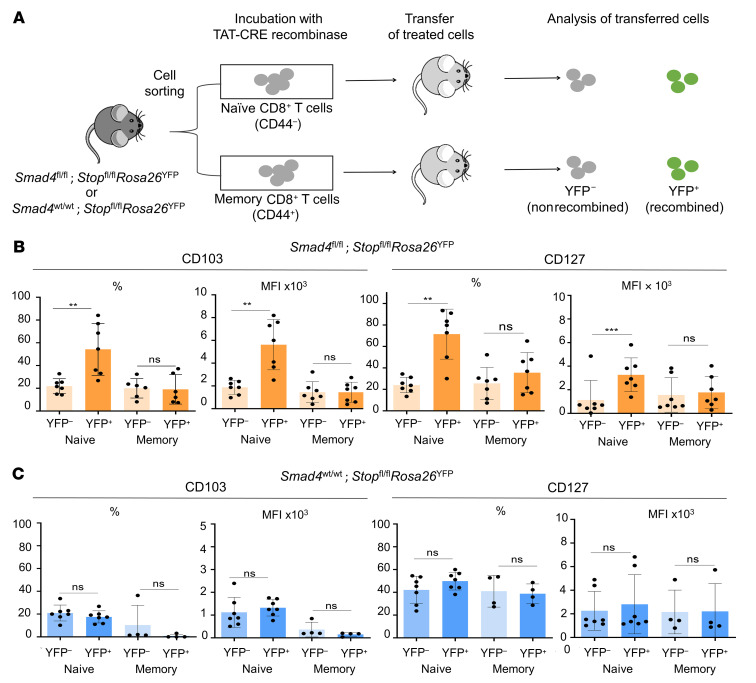
The TGF-β–independent function of SMAD4 that restrains CD103 and IL-7R occurs in CD8^+^ T cells at the naive stage. (**A**) Experimental procedure for TAT-CRE experiment. Naive and memory CD8^+^ T cells were purified from either *Smad4^fl/fl^* *Stop^fl/fl^* *Rosa26*^EYFP^ or *Smad4^wt/wt^* *Stop^fl/fl^* *Rosa26*^EYFP^ mice and treated with TAT-CRE recombinant protein prior to transfer into RAG2-KO mice and 3 weeks later, cells were recovered and analyzed by flow cytometry. (**B**) Histograms illustrating the expression of CD103 and CD127 in YFP^+^ (recombined) in the naive (*n* = 7) or memory state (*n* = 6) CD8^+^ T cells and YFP^–^ (nonrecombined) counterpart CD8^+^ T cells from *Smad4^fl/fl^* *Stop^fl/fl^* *Rosa26*^EYFP^ mice. (**C**) Similarly, histograms depicting the expression of CD103 and CD127 in YFP^+^ in the naive (*n* = 7) or memory state (*n* = 4) CD8^+^ T cells and YFP^–^ counterpart CD8^+^ T cells from *Smad4^wt/wt^* *Stop^fl/fl^* *Rosa26*^EYFP^ mice. The data in panels **B** and **C** are expressed as percentage or MFI. Each symbol represents an individual mouse and *n* = 4 or more for each group. All data represent at least 3 independent experiments and are presented as mean ± SD. Data were analyzed by a paired Student’s *t* test. **P < 0.01, ***P < 0.001. NS, not significant (*P* > 0.05).

We then sought to delineate at which stage of CD8^+^ T cell differentiation SMAD4 conditions CD8^+^ T cells. To achieve this, we selectively deleted SMAD4 in purified naive versus memory/experienced CD44^+^ CD8 T cells from *Smad4^fl/fl^* *Stop^fl/fl^* *Rosa26*^YFP^ or *Smad4^wt/wt^* *Stop^fl/fl^* *Rosa26*^YFP^ mice, upon brief treatment with the membrane-permeant recombinant TAT-CRE protein (27, 28). The TAT-CRE–treated CD8^+^ T cells were then transferred into mice (Figure 9A). The analysis of YFP^+^ cells (recombined cells), 3 weeks after transfer, showed that the loss of SMAD4 at the naive stage significantly increased CD103 and IL-7R expression on CD8^+^ T cells, as described in SKO animals compared to their YFP^–^ counterparts, which have SMAD4. Interestingly, SMAD4 ablation in memory/experienced CD8^+^ T cells did not lead to an increase in surface expression of CD103 or IL-7R (Figure 9B). Similarly, TAT-CRE treatment of the naive or memory CD8^+^ T cells from the control *Smad4^wt/wt^* *Stop^fl/fl^* *Rosa26*^YFP^ mice did not result in an upregulation of CD103 or IL-7R surface expression (Figure 9C). Given these results, it appears that SMAD4 represses the expression of these markers at the naive stage and that it is only at this stage that specific deletion of SMAD4 can lead to their aberrant overexpression. Overall, our findings reveal that CD8^+^ T cells are conditioned by TGF-β–independent SMAD4 function primarily during the naive stage rather than once the CD8^+^ T cells are activated/memory.

### In the absence of TGF-βR engagement, SMAD4 mediates epigenetic control of TGF-β target genes and regulates their expression in naive CD8^+^ T cells.

**Figure 10:**
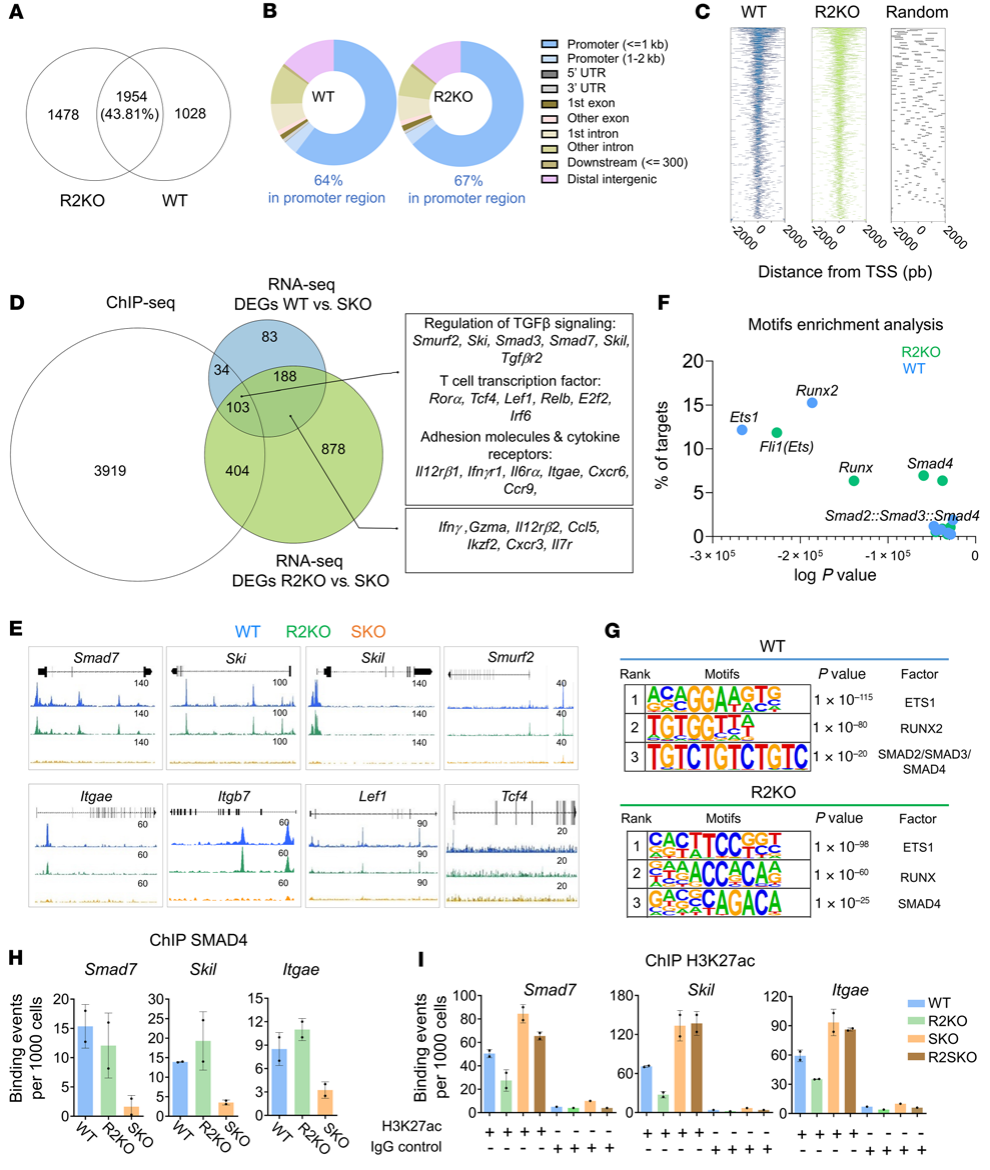
In the absence of TGF-βR signaling, SMAD4 largely binds to DNA and mediates epigenetic control of TGF-β target genes in naive CD8^+^ T cells. (**A**) Venn diagram showing the number of SMAD4 common peaks between WT (pool of 3 mice) and R2KO (pool of 5 mice) naive CD8αβ^+^ T cells. (**B**) The proportions of SMAD4 peaks associated with promoter, 5′ UTR, 3′ UTR, exon, intron, and intergenic regions in WT and R2KO naive F5 CD8αβ^+^ T cells. (**C**) Enriched heatmaps showing the SMAD4 occupancy signals in genomically aggregated TSS regions in WT and R2KO CD8^+^ T cells. Each panel represents 2 kb upstream and downstream of the TSSs. (**D**) Venn diagram showing the overlap between SMAD4 ChIP-seq peaks and RNA-seq DEGs. (**E**) SMAD4-binding ChIP-seq peaks in WT (blue), R2KO (green), or SKO control (orange), in corresponding genes. (**F**) Transcription factor (TF) top motifs in SMAD4-binding sites in WT and R2KO CD8^+^ T cells. The *x* axis represents the log(*P* value) of the motif enrichment, and the *y* axis represents the fold change of the motif enrichment. (**G**) The 3 top motifs found by hypergeometric optimization of motif enrichment (HOMER) analysis among SMAD4-binding peaks in WT and R2KO CD8^+^ T cells. (**H**) qPCR-based ChIP analysis of SMAD4 on the promoters/enhancers of *Smad7*, *Skil*, and *Itgae* in WT, R2KO, and SKO F5 naive CD8αβ^+^ T cells. Each data point represents a pool of 3–5 mice. (**I**) qPCR-based ChIP analysis of H3K27ac on the promoters/enhancers of *Smad7*, *Skil*, and *Itgae* in WT, R2KO, SKO, and R2SKO F5 naive CD8αβ^+^ T cells. Each data point represents a pool of 3–5 mice.

To further decipher at the chromatin level the mechanisms by which SMAD4 regulates TGF-β signature imprinting in naive CD8^+^ T cells, prior to any TGF-β signal, we conducted chromatin immunoprecipitation combined with sequencing (ChIP-seq) on SMAD4 from naive CD8^+^ T cells from WT, R2KO, and SKO mice. A total of 2982 peaks were identified in WT cells and 3432 peaks were identified in R2KO cells, demonstrating that SMAD4 broadly binds to the genome even without TGF-β signaling in naive CD8^+^ T cells. Of the 2982 peaks in WT cells and 3432 peaks in R2KO cells, 1954 peaks were common, highlighting an important similarity in regional binding sites irrespective of the cellular response to TGF-β (Figure 10A). Since most of the binding sites were localized in promoter regions (64% for WT and 67% for R2KO) or were closely located around the transcription start site (TSS) regions (Figure 10, B and C, and Supplemental Figure 13A), this indicates that SMAD4, before any TGF-βR engagement, occupies promoters and enhancers of many genes in naive CD8^+^ T cells and may regulate their expression. Interestingly, SMAD4 binds irrespective of TGF-β signaling to genomic regions that regulate diverse biological pathways involved for instance in TCR signaling, RNA translation, or TGF-β signaling regulation in naive CD8^+^ T cells (Supplemental Figure 13B). These data highlight the broad potential impact of the TGF-β–independent function of SMAD4 in diverse CD8^+^ T cell biological processes already at the naive stage.

In order to associate this strategic location and direct gene regulation, we combined the ChIP-seq peaks of SMAD4 and the DEGs from the RNA-seq (Figure 4). We found that more than one-third of the DEGs (541 genes) contain SMAD4 binding sites and are potentially directly regulated by SMAD4 in CD8^+^ T cells (Figure 10D). Among those DEGs potentially directly regulated by SMAD4, 103 genes were differentially expressed regardless of TGF-β signal. Those SMAD4 TGF-β–independent genes comprised genes implicated in CD8^+^ T cell differentiation, such as *Tcf4* and *Lef1*, but also many well-characterized TGF-β target genes. More precisely, we found TGF-β repressors (*Smad7*, *Smurf2*, *Ski*, and *Skil*) and genes involved in lymphocyte epithelial retention (e.g., *Itgae*), the expression of which is upregulated upon SMAD4 deficiency (Figure 10, D and E, and Supplemental Figure 13C). Thus, SMAD4, by acting at the chromatin level, directly impedes TGF-β target gene expression in CD8^+^ T cells, before any TGF-βR engagement. To identify putative partners of SMAD4 in WT and R2KO CD8^+^ T cells, we conducted an enrichment motif analysis. We found similar motifs in the top 3 enriched motifs, notably ETS and RUNX family motifs (Figure 10, F and G). Interestingly, this indicates a potential interaction between SMAD4 and ETS or RUNX transcription factors that are important regulators of T cell differentiation and homeostasis (29, 30), exemplifying that SMAD4 interacts with different partners to mediate its wide transcriptional impact in CD8^+^ T cells. Notably, BMP and activin, other members of the TGF-β family that signal through SMAD4, have been proposed to alter the biology of CD4^+^ T cells (31, 32). However, treatment of either naive CD8^+^ T cells or activated CD8^+^ T cells with either BMP or activin failed to enforce the TGF-β–independent function of SMAD4 and alter the ability of TGF-β to induce CD103 expression (Supplemental Figure 14, A and B). These observations suggest that BMP and activin are not the extracellular signals that mediate the TGF-β–independent SMAD4 effects in CD8^+^ T cells.

Finally, to gain further insight into the epigenetic mechanisms by which SMAD4 mediates TGF-β target gene repression before TGF-β signaling, we performed ChIP of SMAD4 and ChIP of a histone mark associated with gene expression, namely the acetylation of the 27th lysine residue of the histone H3 protein (H3K27ac). We found that H3K27ac was enriched at the same SMAD4 binding regions of *Smad7*, *Skil*, and *Itgae*, specifically in the absence of SMAD4 (SKO and R2SKO CD8^+^ T cells). In contrast, we observed less enrichment in these SMAD4 binding regions in R2KO CD8^+^ T cells (Figure 10, H and I). This indicates that SMAD4, in a TGF-β–independent manner and opposite to TGF-βR signaling, promotes histone deacetylation of TGF-β target gene promoters and enhancers, likely mediating their repression.

## Discussion

Our study uncovers a fundamental role for a TGF-β–independent function of SMAD4 in preconditioning CD8^+^ T cell fate, thereby limiting IBD development. We reveal that the TGF-β–independent function of SMAD4 acts as a basal repressor of TGF-β target genes, restraining the TGF-β signature in CD8^+^ T cells in absence of any TGF-βR engagement. Although SMAD4 in T cells promotes naive CD8^+^ T cells with effector predisposition, it prevents their accumulation and intestinal epithelial retention. In addition, SMAD4 represses the expression of genes encoding components of the TGF-β negative feedback loop in an anticipatory manner. Thus, we propose that SMAD4 contributes to predisposing CD8^+^ T cells to respond to the immune-regulatory effects of TGF-β. Subsequently, upon SMAD4 deprivation, CD8^+^ T cells accumulate, and are retained in the intestinal epithelium where they escape from TGF-β control and can be chronically activated by the microbiota.

Pioneering works have reported that SMAD4 ablation in T cells promotes either Th2 or Th17 development and contributes to the development of gastrointestinal cancer lesions in mice at an advanced age (14, 15). In the present study, we focused on deciphering mechanisms that contribute to the establishment of the chronic intestinal inflammation and we performed our analysis at an earlier time point compared with the aforementioned works. Specifically, our data reveal the importance of CD8α^+^ T cells and TGF-β–independent SMAD4 effects in controlling CD8αβ^+^ T cell accumulation, localization, and pro-pathogenic potential within the intestine preventing the development of severe chronic inflammation. The strategical epithelial location of CD8αβ^+^ T cells and not that of CD4^+^ T cells, which are largely located in the lamina propria, may enable CD8^+^ T cells to initiate damage to the epithelium in a microbiota-dependent manner, which leads to subsequent inflammation. In line with this scenario, in different IBD models, CD8^+^ T cells were proposed to kill intestinal epithelial cells, contributing in different ways to initiate chronic inflammation (33, 34). Furthermore, in humans, growing evidence suggests that the accumulation and aberrant activation of CD8^+^ T cells within the intestinal mucosa correlates with IBD (19, 35). The mechanisms underlying CD8^+^ T cell activation in IBD remain unclear, however. In the present study, we uncover that the microbiota plays a central role in driving CD8^+^ T cell activation. Whether a specific strain of bacteria is required to cause CD8^+^ T cell activation or whether any strain of bacteria can do so is unclear. Furthermore, the mechanisms by which the bacteria cause epithelial accumulation and activation of CD8^+^ T cells have yet to be determined. Indeed, microbiota can promote CD8^+^ T cell recruitment and activation in various ways, including possibilities of creating antigenic, metabolic, and inflammatory niches in the gut that support CD8^+^ T cell activation (36, 37).

Our current results reveal that independently of TGF-β, SMAD4 promotes CD8^+^ T cell activation by predisposing an effector differentiation program in naive CD8^+^ T cells. It has been reported that SMAD4 contributes to T cell activation by inducing c-Myc during T cell activation (17). Therefore, additional deletion of SMAD4 in TGF-βR–KO T cells prevents autoimmunity. Here, we reveal that SMAD4-promoted T cell activation intervenes prior to any TCR-dependent activation. Our findings enforce studies questioning the previously presented dogma by which naive T cells are considered unpoised (38). Indeed, depending on their developmental origin, naive CD8^+^ T cells could be differentially “preconditioned,” thereby imprinting their fate after peripheral cognate antigen encounter (39). Our study describes SMAD4 as a critical actor of this preconditioning and future investigation will be required to determine the molecular partners of SMAD4 that are important in mediating this process in naive CD8^+^ T cells.

In seminal work, Massagué et al. demonstrated the importance of TGF-β in controlling T cell effector gene expression (40). The exact signaling branch of TGF-β, critical in establishing TGF-β–driven immunosuppression, remains nevertheless imprecise. Here, we reveal that the absence of SMAD4 reduced CD8^+^ T cell sensitivity to TGF-β. Indeed, absence of SMAD4 elicits CD8^+^ T cell activation in such a manner that even genetic activation of the remaining TGF-β pathways cannot rescue TGF-β–mediated immunosuppression. Interestingly, this poor response to TGF-β is reminiscent of what is described in patients suffering from IBD. Due to an elevated expression of SMAD7, a potent TGF-β signaling repressor, T cells from those patients are not responsive to the immunoregulatory effect of TGF-β (12). Thus, the poor effector predisposition that results from deletion of SMAD4 is overcome by escape from the immunosuppressive effect of TGF-β, thereby facilitating microbiota-driven chronic CD8^+^ T cell activation.

TGF-β is critical for CD103 induction and IEL formation and retention in the gut (26, 41). In a recent study, it was reported that SMAD4 restrains the generation of CD103^+^CD8^+^ tissue-resident memory T cells after viral infection in the lungs (42). However, the role of SMAD4 in limiting the microbiota-mediated generation of intestinal CD8αβ^+^ IELs and its consequences for IBD have not been investigated to the best of our knowledge. In addition, we reveal that the TGF-β–independent function of SMAD4 epigenetically restrains the epithelial retention ability of CD8^+^ T cells, already at the naive stage. TGF-βRII–KO (R2KO) mice did not develop severe intestinal inflammation, in contrast to TGF-βRII and SMAD4 double-KO mice (R2SKO). This difference is likely due to the lack of expression of CD103 in CD8^+^ T cells from R2KO mice. Indeed, ablation of SMAD4 in a setting of TGF-βRII deficiency restores the epithelial retention capacity of CD8^+^ T cells. This restoration could explain the severe intestinal immunopathology observed in R2SKO compared with R2KO mice. Accordingly, CD103 blockade alleviates intestinal immunopathology in both SMAD4-deficient mice and human patients with Crohn’s disease (43), pinpointing the central role of gut epithelial retention of CD8^+^ T cells in IBD development.

SMAD4 deletion and total TGF-β signaling disruption have strikingly opposite transcriptional and functional outcomes in CD8^+^ T cells. We show that SMAD4 binds to promoter regions of numerous TGF-β target genes and regulates their expression in the absence of TGF-β signaling by inducing epigenetic modifications such as chromatin acetylation. By repressing several TGF-β repressors such as *Smad7*, *Ski*, *Skil*, and *Smurf2*, SMAD4 facilitates TGF-β effects, in a TGF-β–independent manner. In line with this potentiation, SMAD4 limits the basal expression of TGF-β target genes and likely allows better fine-tuned regulation of T cell responses to TGF-β. Indeed, by reducing the background (“noise”) expression of TGF-β target genes, in the absence of TGF-β signal, SMAD4 potentiates the efficient action of TGF-β once a TGF-β signal is received. This uncharacterized anticipatory regulation governed by SMAD4 in CD8^+^ T cells, in a TGF-β–independent manner, explains the dual effect by which SMAD4 restrains the action of TGF-β prior to TGF-β signaling, but potentiates it after TGF-βR engagement.

Further investigations will be necessary to determine how SMAD4 operates independently of TGF-β in CD8^+^ T cells and uncover its partners in mediating this critical and nonredundant TGF-β–independent function. Our data suggest that BMPs and activin are unlikely to be the extracellular signal mediating SMAD4 TGF-β–independent functions. Notably, a previous study reported that SMAD4 can translocate to the nucleus without any extracellular signal (44), suggesting that this possibility may also exist in naive CD8^+^ T cells. In the present study, we found that transcription factors with either an ETS or a RUNX DNA-binding domain might function with SMAD4 to mediate its TGF-β–independent function in naive CD8^+^ T cells. In CD4^+^ T cells, SMAD4 mediates the repression of the *Rorc* locus by interacting with SKI, thereby inhibiting Th17 generation (18). In NK cells, JUNB interacts directly with SMAD4 to promote GZMB expression (45). Thus, the wide transcriptional shift observed upon SMAD4 ablation likely results from its interactions with several distinct partners, regulating specific genes according to the molecular context, which may change depending on the activation status of the CD8^+^ T cells. In line with this contextual effect of SMAD4, we show that ablation of SMAD4 in naive CD8^+^ T cells versus activated/memory CD8^+^ T cells does not result in a similar phenotype.

In summary, our study reveals that SMAD4 preconditions the fate of naive CD8^+^ T cells. We uncover a critical and nonredundant regulation governed by SMAD4 that, in a TGF-β–independent manner, finely preprograms naive CD8^+^ T cell homeostasis, with direct consequences for chronic intestinal inflammation.

## Methods

### Mice.

*CD4*-Cre *Smad4^fl/fl^* (SKO), *CD4*-Cre *Trim33^fl/fl^* (TKO), *CD4*-Cre *Smad4^fl/fl^* *Trim33^fl/fl^* (STKO), *CD4*-Cre *Smad4^fl/fl^* *Tgfbr2^fl/fl^* (R2SKO), *CD4*-Cre *Tgfbr2^fl/fl^* (R2KO), and *CD4*-Cre *Stop^fl/fl^* *Tgfbr1^CA^* (RCA) mice were generated as previously described (23, 46). SKO-RCA mice were obtained by crossing *CD4*-Cre *Smad4^fl/fl^* with *Stop^fl/fl^* *Tgfbr1^CA^* mice. *Stop^fl/fl^* *Tgfbr1^CA^* mice were provided by Laurent Bartholin (Cancer Research Center Lyon, Lyon , France). *Smad4^fl/fl^ Stop^fl/fl^* *Rosa26*^YFP^ mice were obtained by crossing *Smad4^fl/fl^* mice with *Stop^fl/fl^* *Rosa26*^YFP^ mice (47). RAG2-KO mice were obtained from Charles River Laboratories. The different F5 transgenic mouse models were obtained by crossing SKO, or R2SKO and R2KO mice with F5 transgenic mice in a RAG2-KO background (22). F5 mice were provided by Dimitris Kioussis (National Institute for Medical Research, London, United Kingdom). All mice were on the C57BL/6J background and were maintained in Anican/P-PAC, a specific pathogen–free animal facility of the CRCL, Lyon, France. Unless mentioned otherwise, male and female mice were used.

### BM transfer, CD8^+^/CD4^+^ T cell depletion, and CD103 blockade.

RAG2-KO mice were irradiated (6 Gy) and reconstituted by intraorbital injections with 1 × 10^6^ T cell–depleted BM cells from WT, R2KO, SKO, or R2SKO mice. For the CD8^+^/CD4^+^ T cell depletion, 20 days after BM reconstitution, mice intraperitoneally received 150 μg of anti-CD8β (clone 53-5.8), anti-CD4 (clone GK1.5), rat IgG1 isotype control (clone HRPN), or rat IgG2b isotype control (clone LTF-2) once per week. We used different antibody clones to verify the depletion by flow cytometry. For CD103 blockade, RAG2-KO mice were irradiated (6 Gy) and reconstituted by intraorbital injections with 1 × 10^6^ T cell–depleted BM cells either from WT or SKO mice and 14 days after BM reconstitution, and mice intraperitoneally received 100 μg of anti-CD103 blockade antibody (clone M290) or PBS 3 times per week. All the antibodies used for in vivo experiments were obtained from BioXCell.

### Histological assessment of inflammation.

Colon and small intestine were fixed in 2% formaldehyde (VWR), embedded in paraffin, and sectioned. Hematoxylin and eosin (H&E; Sigma-Aldrich) staining was performed in embedded tissue. Intestinal inflammation was scored in a blinded fashion using a scoring system based on the following criteria: colon length, inflammatory cell infiltrate (severity and extent), crypt hyperplasia, and presence of neutrophils within the crypts, presence of crypt abscesses, erosion, granulation tissues, and villous blunting.

### Antibiotic treatment.

For antibiotic treatment, drinking water was supplemented with an antibiotic cocktail composed of ampicillin (1 g/L), metronidazole (1 g/L), neomycin (1 g/L), and vancomycin (0.5 g/L), all purchased from Sigma-Aldrich. Antibiotics were administered after weaning and until 5 months of age.

### Spleen, lymph node, lung, skin, and lamina propria cell isolation.

Spleens, peripheral lymph nodes (inguinal and axillary), and MLNs were dissociated using a nylon mesh and red blood cells were lysed with 9 g/L NH_4_Cl complemented vol/vol with culture medium. Lungs and ears were cut in small pieces and incubated in RPMI medium (Gibco) containing 20% fetal bovine serum (Gibco), 100 μg/mL DNase I (Roche), and 1 mg/mL collagenase from *Clostridium* *histolyticum* (Sigma-Aldrich). For lungs, mice were perfused with PBS, filtered, and centrifuged in a 44%–67% Percoll gradient. Small and large intestines were dissected after removing fat and Peyer’s patches. Intestines were longitudinally opened and washed in PBS. Intestines were cut into small pieces and incubated with 5 mM EDTA and 1 mM DTT (Sigma-Aldrich) at 37°C under agitation. Epithelial cells and IELs were separated from tissue after 20 minutes. Tissues were then digested in RPMI medium containing 20% fetal bovine serum, 100 μg/mL DNase I, and 0.6 mg/mL collagenase. Intestinal lamina propria was harvested from a 44%–67% Percoll gradient run for 20 minutes at 1300*g* and 20°C.

### Flow cytometry.

Cells prepared as described above were stained with a yellow viability marker and blocked using FcR blocking reagent and then stained with antibodies (Table 1). For cytokine staining, cells were stimulated ex vivo for 4 hours with 1 μg/mL PMA and 1 μg/mL ionomycin (both Sigma-Aldrich) in the presence of brefeldin A and GolgiStop (BD Biosciences). After extracellular staining, cells were fixed and permeabilized with a Cytofix/Cytoperm kit (BD Biosciences) and then stained with antibodies directed against IFN-γ, TNF-α, CCL3, and IL-17A. GZMA, GZMB, TBET, FOXP3, and RORγt staining was performed after cells were fixed and permeabilized with an eBioscience Foxp3/Transcription Factor Staining Buffer Set. For p-STAT5 intracellular staining, cells were fixed with 2% paraformaldehyde (EMS Company) for 10 minutes at room temperature and then permeabilized with ice-cold methanol for 30 minutes before intracellular staining. Flow cytometry data were acquired on an LSR Fortessa using DIVA software and analyzed by FlowJo software (all from BD Biosciences).

### RT-PCR.

RNA was isolated with the RNeasy mini kit (Qiagen) and reverse transcribed using an iScript cDNA synthesis kit (Bio-Rad). Quantitative RT-PCR was performed using LightCycler 480 SYBR Green Master (Roche) and different sets of primers on a LightCycler 480 Real-Time PCR System (Roche). Samples were normalized to *Gadph* and analyzed according to the ΔΔCt method. Primer sequences are provided in Table 2.

### Bioinformatic analyses.

All genomic data were analyzed with R version 3.6.3 (https://cran.r-project.org/) and Bioconductor (http://www.bioconductor.org/.).

### RNA-seq.

Illumina sequencing was performed on RNA extracted from triplicates of each condition. Standard Illumina bioinformatics analyses were used to generate fastq files, followed by quality assessment, trimming, and demultiplexing. Rsubread v1.34.6 was used for mapping to the hg38 genome and creating a matrix of RNA-seq counts. Next, a DGEList object was created with the edgeR package v3.26.7 (48). After normalization for composition bias, gene-wise exact tests were computed for differences in the means between groups, and DEGs were extracted based on an FDR-adjusted *P* value of less than 0.05. GSEA was performed using the Molecular Signature Database of the Broad Institute (49). To combine 2 data sets from different batches, count data from the 2 data sets were merged and the batch reference was included as a term in the EdgeR linear model without data transformation. For purposes of visualization (heatmaps), the limma package function removeBatchEffect was applied to the log-transformed counts per million (cpm) values before plotting.

### ChIP-seq.

ChIP libraries were prepared and sequenced (Illumina NextSeq 500) using a standard workflow (Active Motif). Resulting 75-nt single-end reads were mapped to the mm10 genome using the BWA algorithm with default settings (50). Only reads that passed Illumina’s purity filter, aligned with no more than 2 mismatches, and mapped uniquely to the genome were used in subsequent analyses. In addition, duplicate reads were removed. After normalization, the peak callers MACS/MACS2 (51) were used to describe genomic regions with local enrichments in tag numbers relative to the input data file (random background). Genomic ranges (GenomicRanges package) were used to perform genomic context annotations using the R packages annotatr (52), ChIPSeeker (53), and ChipPeakAnno (54). Enriched heatmaps (EnrichedHeatmap) (55) were used to visualize average ChIP peak signals. HOMER v3.12 (http://homer.ucsd.edu/homer/) was used to calculate motif enrichment in the vicinity of ChIP peaks. All sequencing data have been deposited in the NCBI’s Gene Expression Omnibus database (GEO GSE188934). GSE188932 and GSE188933, respectively, are accession numbers for the ChiP-seq and RNA-seq data.

### Human sample in silico analysis.

Human IBD scRNA-seq data sets were reanalyzed from Jaeger et al. and Smillie et al. (19, 20). The data sets were analyzed with the Seurat package v.4.0.1 (56). After filtering for number of features lower than 4000 and percentage of mitochondrial genes lower than 25%, Seurat objects were preprocessed using Seurat functions for normalization (SCTransform), dimension reduction with principal component analysis (PCA) (RunPCA with 30 dimensions), construction of a shared nearest neighbor (SNN) graph (FindNeighbors using 30 dimensions of reduction as input), and clustering (FindClusters with a resolution of 0.5). Selected markers were used to infer Th1 (CD4, TBX21), Th17 (CD4, IL-17A), Th2 (CD4, GATA3), CD8αβ (CD8A, CD8B), and Treg (CD4, IL-2RA) cells.

### Quantitative PCR–based ChIP.

Cells were collected from freshly harvested spleen, MLNs, and peripheral lymph nodes and then sorted using a CD8 Isolation Kit (Miltenyi Biotec, 130-104-075). The PCR-based ChIP was done using a ChIP-It PBMC Kit (Active Motif, 53042) and ChIP-It qPCR Analysis Kit (Active Motif, 53029). ChIP was performed according to the manufacturer’s protocol. IPs were performed using anti-SMAD4 (Abcam, EP618Y), anti-H3K27ac (Abcam, 4729), and rabbit IgG (Cell Signaling Technology, 2729s). Primer sequences are provided in Table 2.

### In vitro survival assay and IL-7 response.

CD8^+^ T cells were obtained from freshly harvested MLNs from F5 transgenic WT, R2KO, SKO, and R2SKO mice. F5 naive CD8^+^ T cells were cultured in 96-well plates (1 × 10^5^ cells/well) in complete RPMI media with or without recombinant IL-7 at 10 ng/mL for different time points (0, 1, 3, and 5 days). For each time point, cells were washed and stained with a Live/Dead Fixable Dead Cell Stains kit (Life Technologies) according to the manufacturer’s protocol, and fluorescent antibodies against CD8 and CD45. The frequency of surviving CD8^+^ T cells was determined by flow cytometry. For p-STAT5 staining, naive CD8^+^ T cells from the MLNs of F5 transgenic WT, R2KO, SKO, and R2SKO mice were starved for 30 minutes at room temperature and then treated with mouse recombinant IL-7 at 10 ng/mL in RPMI plus 2% fetal bovine serum for 30 minutes at 37°C. After 30 minutes of IL-7 stimulation, cells were immediately prepared for p-STAT5 staining (see *Flow cytometry* section).

### In vitro CD8^+^ T cell activation and differentiation and TGF-β treatment.

Naive CD8^+^ T cells from WT, R2KO, and SKO F5 RAG2-KO mice were activated for 4 days via plate-bound anti-CD3/anti-CD28 antibodies (CD3, clone 145-2C11, catalog 16-0031-86; CD28, clone 37.51, catalog 16-0281-86; both coated with antibodies at 10 μg/mL). The cells were cultured (10^5^ cells/well) in 96-well plates coated with anti-CD3/anti-CD28 in complete RPMI media with the presence of IL-7 at 10 ng/mL for all conditions and with or without human recombinant TGF-β1 (Miltenyi Biotec). Four days later, cells were washed and stained with a viability marker and antibodies against CD45, CD8, GZMB, and TBET. For BMP and activin treatment, 0.5 × 10^6^ splenic naive CD8^+^ T cells from F5 RAG2-KO WT mice were treated with 10 ng/mL BMP2 or activin (Peprotech) with or without 10 ng/mL human recombinant TGF-β1 in complete culture media supplemented with 10 ng/mL IL-7 (Peprotech) at 37°C. After 72 hours of culture, cells were prepared for flow cytometry staining (see *Flow cytometry* section).

### In vitro Smad4 deletion.

CD8^+^ T cells from either *Smad4^fl/fl^ Stop^fl/fl^ Rosa26*^YFP^ mice or *Smad4^wt/wt^ Stop^fl/fl^* *Rosa26*^YFP^ mice were isolated using the Mojosort negative selection kit from BioLegend (catalog 480035). Naive (CD44^–^) and memory/experienced (CD44^+^) CD8^+^ T cells were then sorted on a BD FACSAria II based on CD44 expression. Cells were treated for 30 minutes at 37°C with the membrane-permeant recombinant TAT-CRE protein (Merck, SCR508) at the concentration recommended by the manufacturer in order to obtain approximately 50% efficiency. Directly after TAT-CRE treatment, cells were washed in PBS and 150,000 cells reinjected into RAG2-KO mice. Three weeks after transfer, the analysis of YFP^+^ cells (recombined cells) versus YFP^–^ (nonrecombined) was done by isolating splenic CD8^+^ T cells.

### Statistics.

Statistical analysis was performed using GraphPad Prism. Comparisons among multiple groups were performed using 1-way ANOVA followed by Tukey’s test. Comparisons of a group control with different groups were performed using 1-way ANOVA followed by Dunnett’s test. Data from 2 groups were compared by 2-tailed, unpaired or paired Student’s *t* test. *P* values less than 0.05 were considered statistically significant.

### Study approval.

The experiments were conducted in accordance with the guidelines for animal care of the European Union (ARRIVE) and were validated by the local Animal Ethic Evaluation Committee (CECCAPP) and the French Ministry of Research.

## Author contributions

RI and SMS supervised the work and wrote the manuscript. RI, AG, and SMS conducted the experiments. HHV, CB, and ED performed the bioinformatics analysis. RI, HHV, SMS, AG, NB, DB, and JCM discussed the data and provided conceptual input. SMS and JCM provided financial resources.

## Supplementary Material

Supplemental data

## Figures and Tables

**Table 1:**
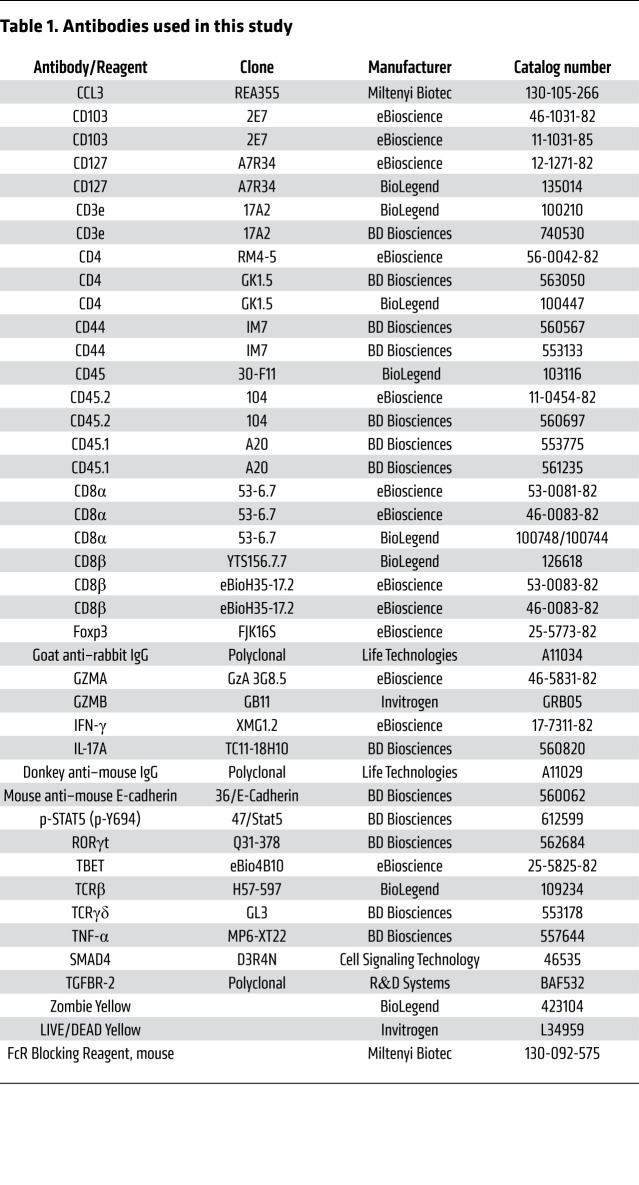
Antibodies used in this study

**Table 2:**
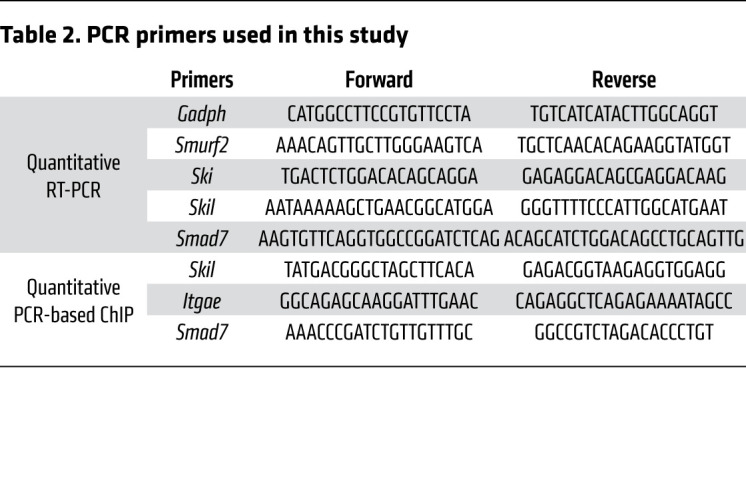
PCR primers used in this study

## Version 1 04/15/2022

Electronic publication
