# Supplementary material for: SMAD4 TGF-β–independent function preconditions naive CD8+ T cells to prevent severe chronic intestinal inflammation
Source: J Clin Invest. 2022 Apr 15;132(8):e151020. doi: 10.1172/JCI151020 (PMC9012287; doi:10.1172/JCI151020)
Supplement: Supplemental data [file jci-132-151020-s024.pdf]

A

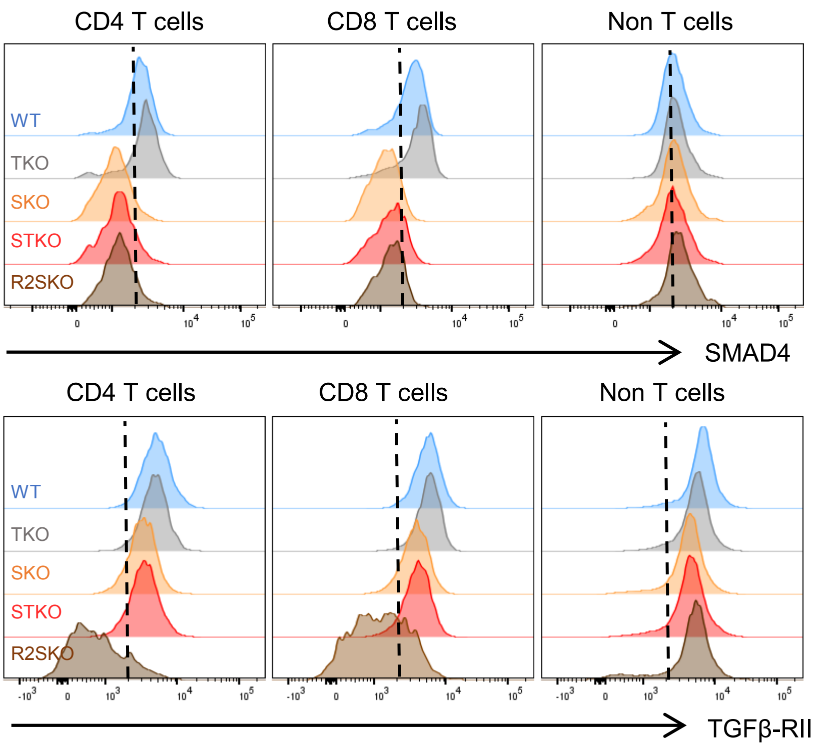
CD4 T cells
CD8 T cells
Non T cells
WT
TKO
SKO
STKO
R2SKO
SMAD4
TGFβ-RII

B

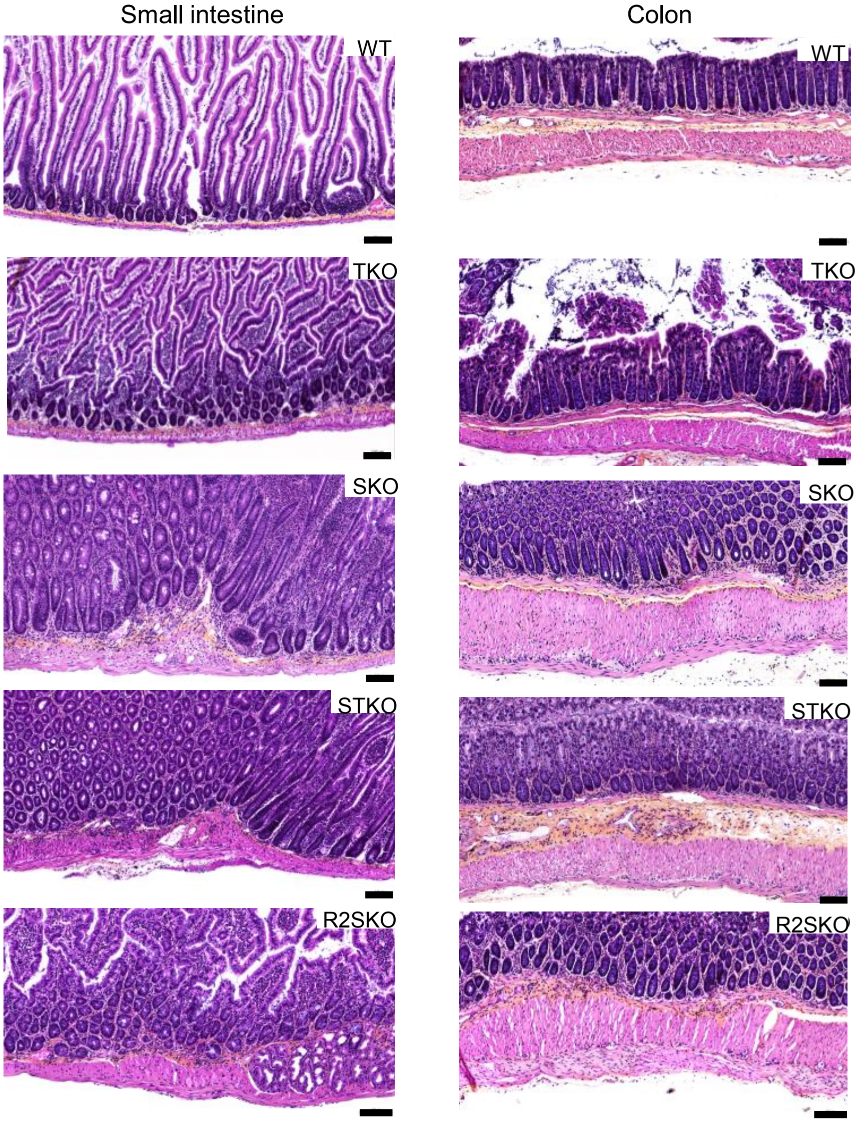
Small intestine
Colon
WT
TKO
SKO
STKO
R2SKO

Supplemental Figure 2

A

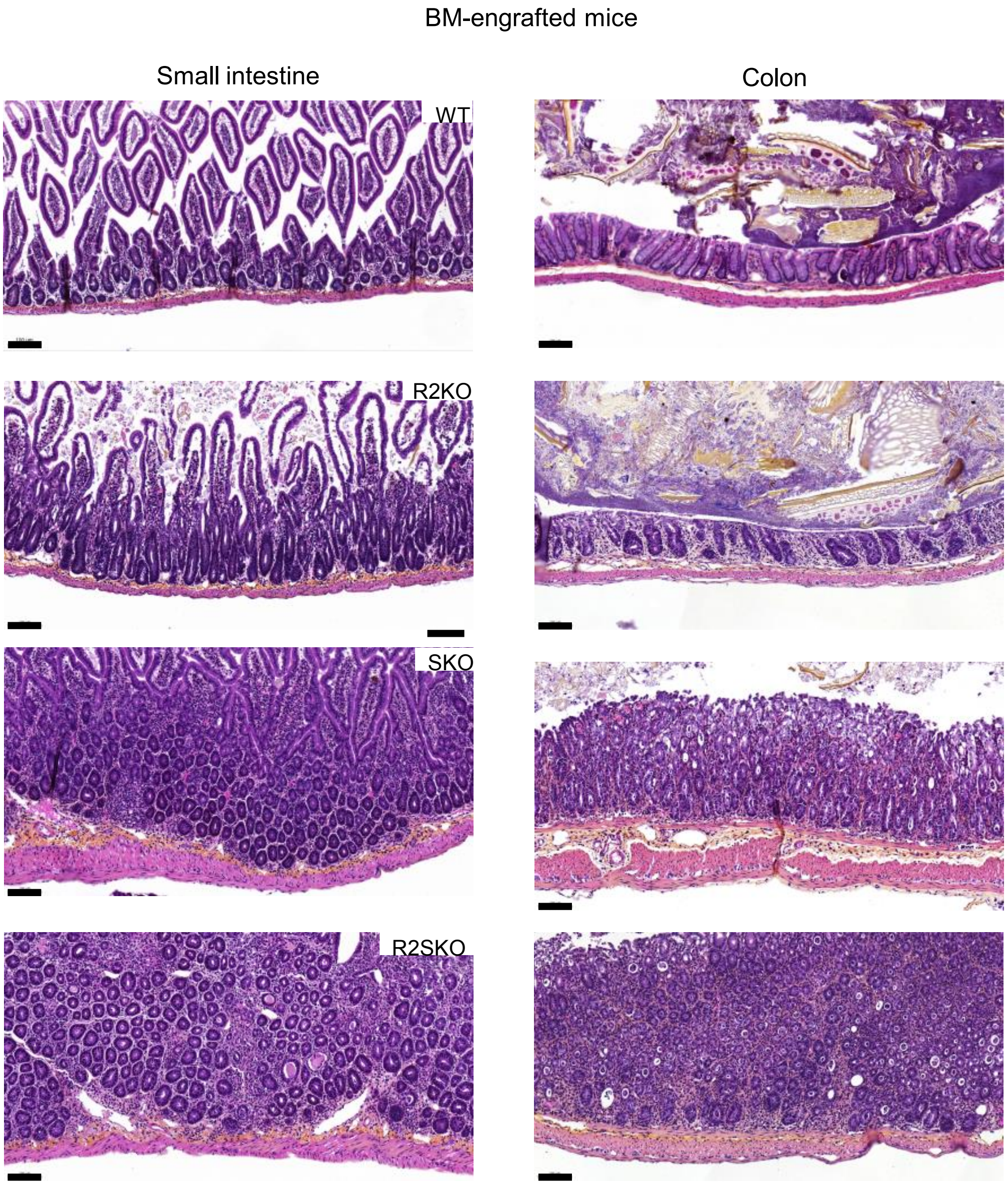

Supplemental Figure 3

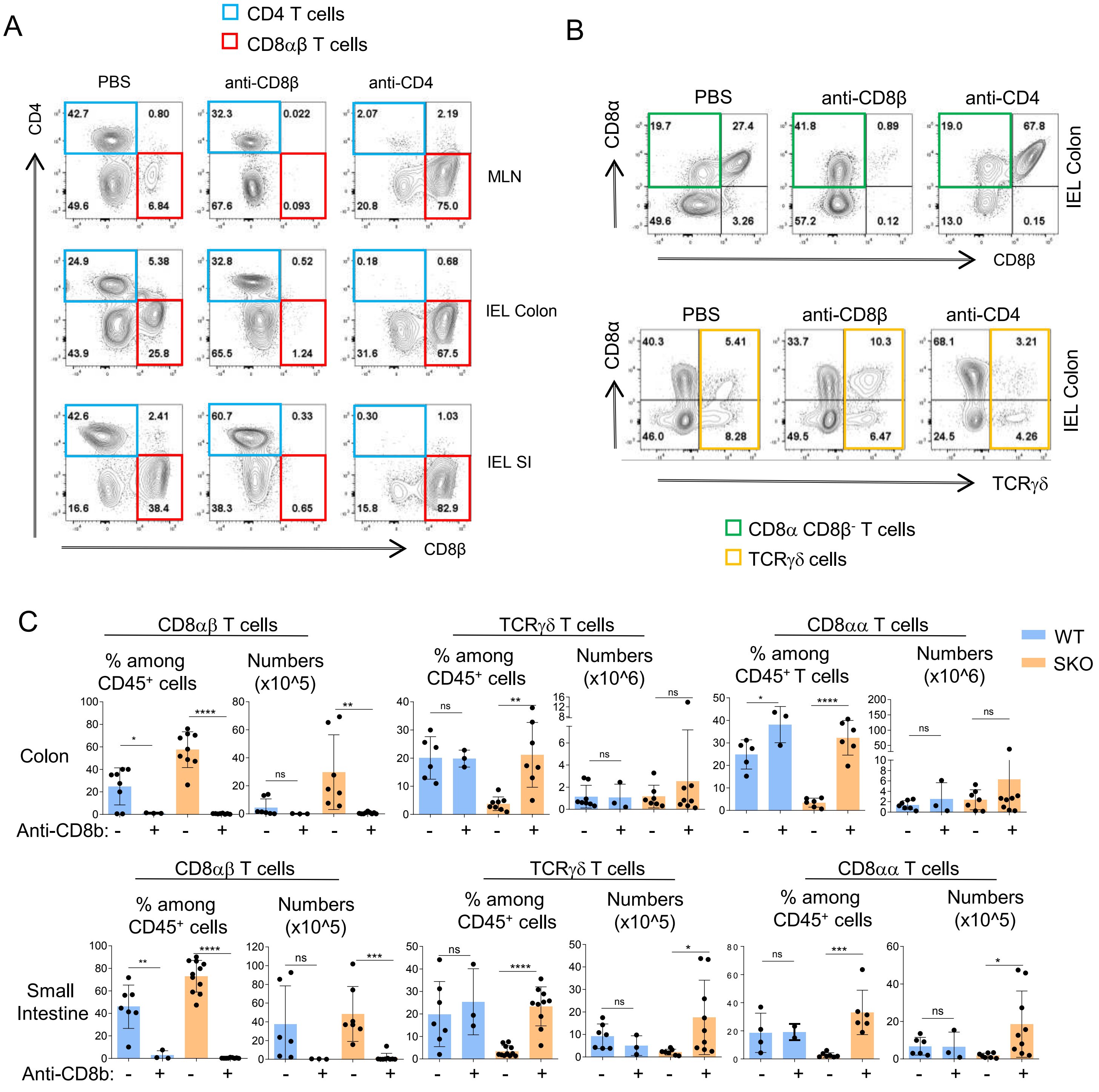

Supplemental Figure 4

A

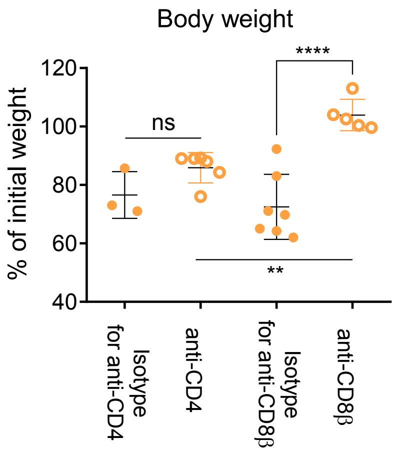

B

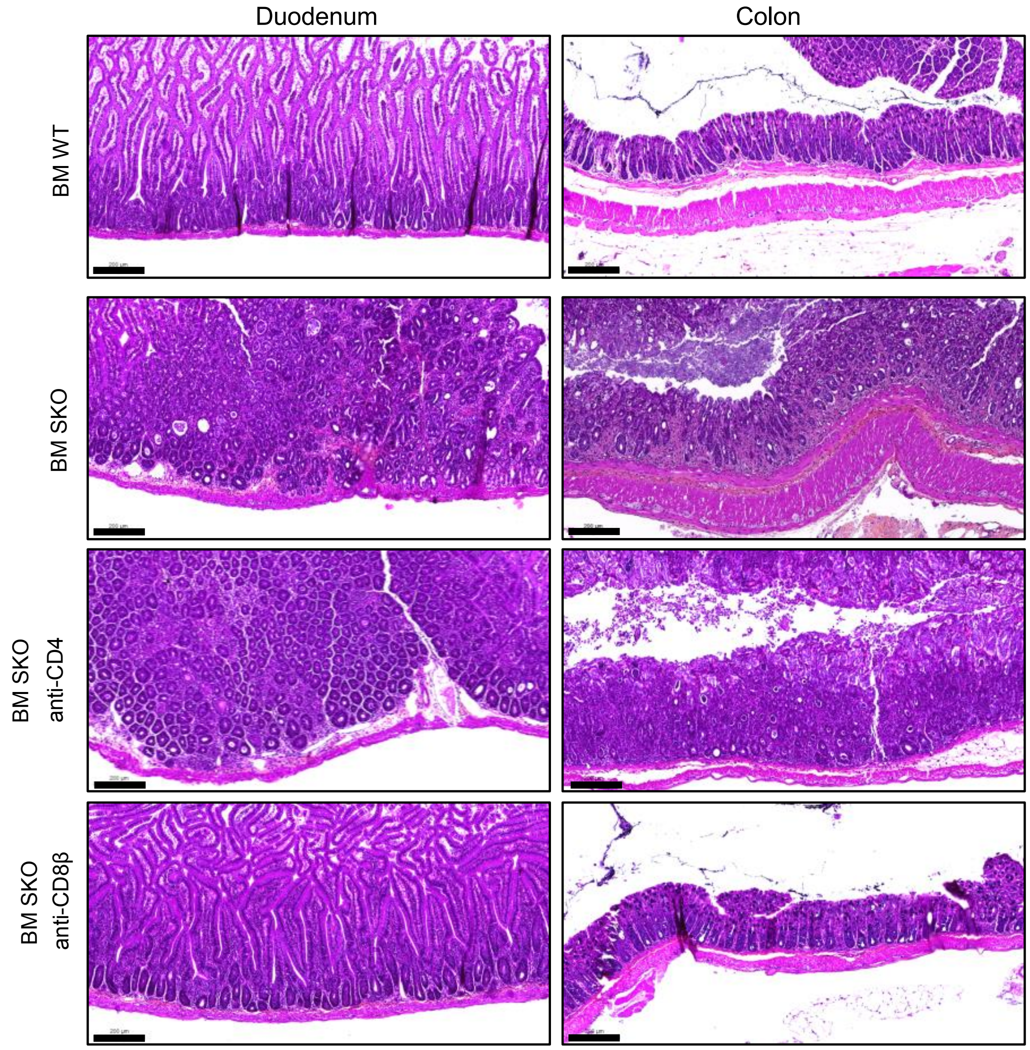

C

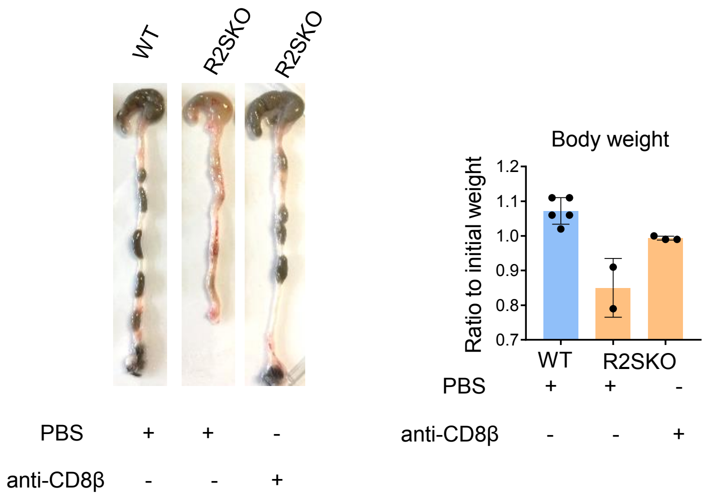

Supplemental Figure 5

A

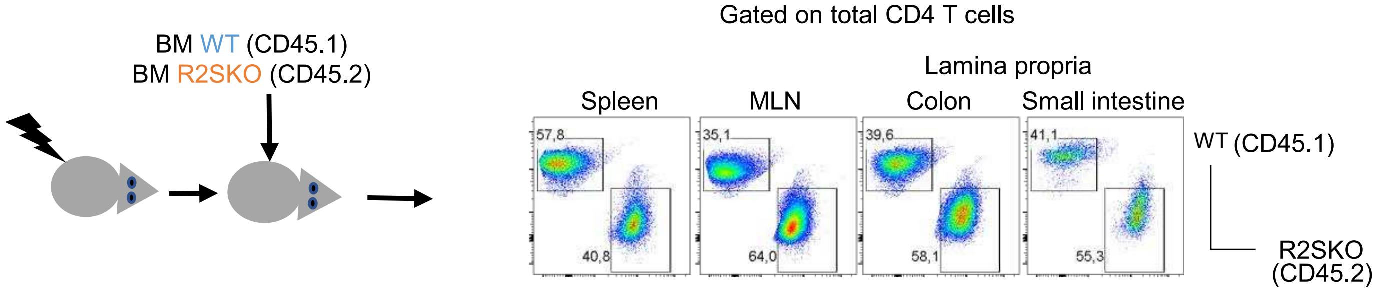

B

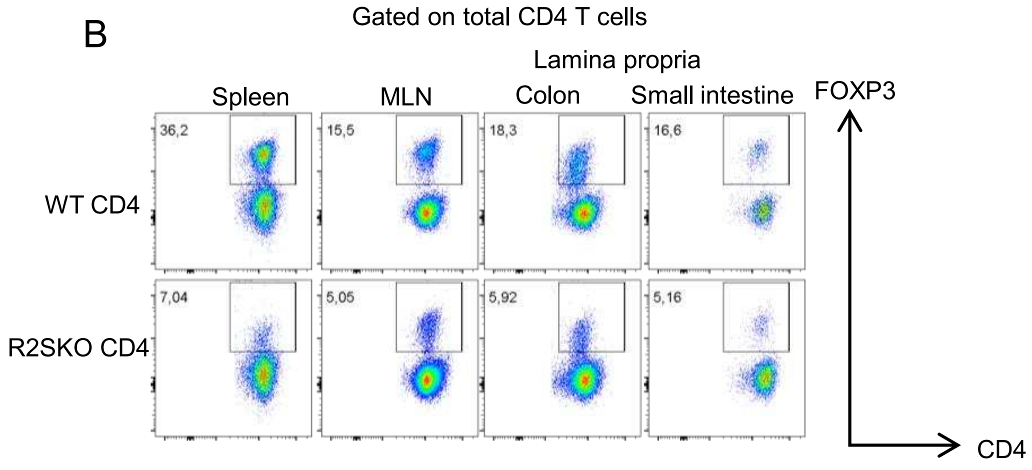

C

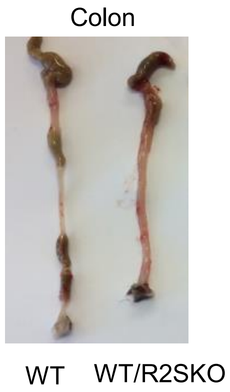

C

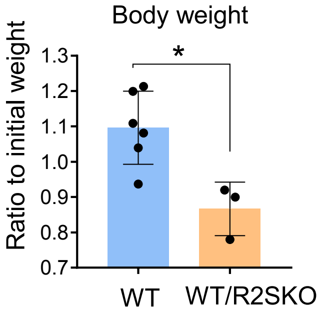

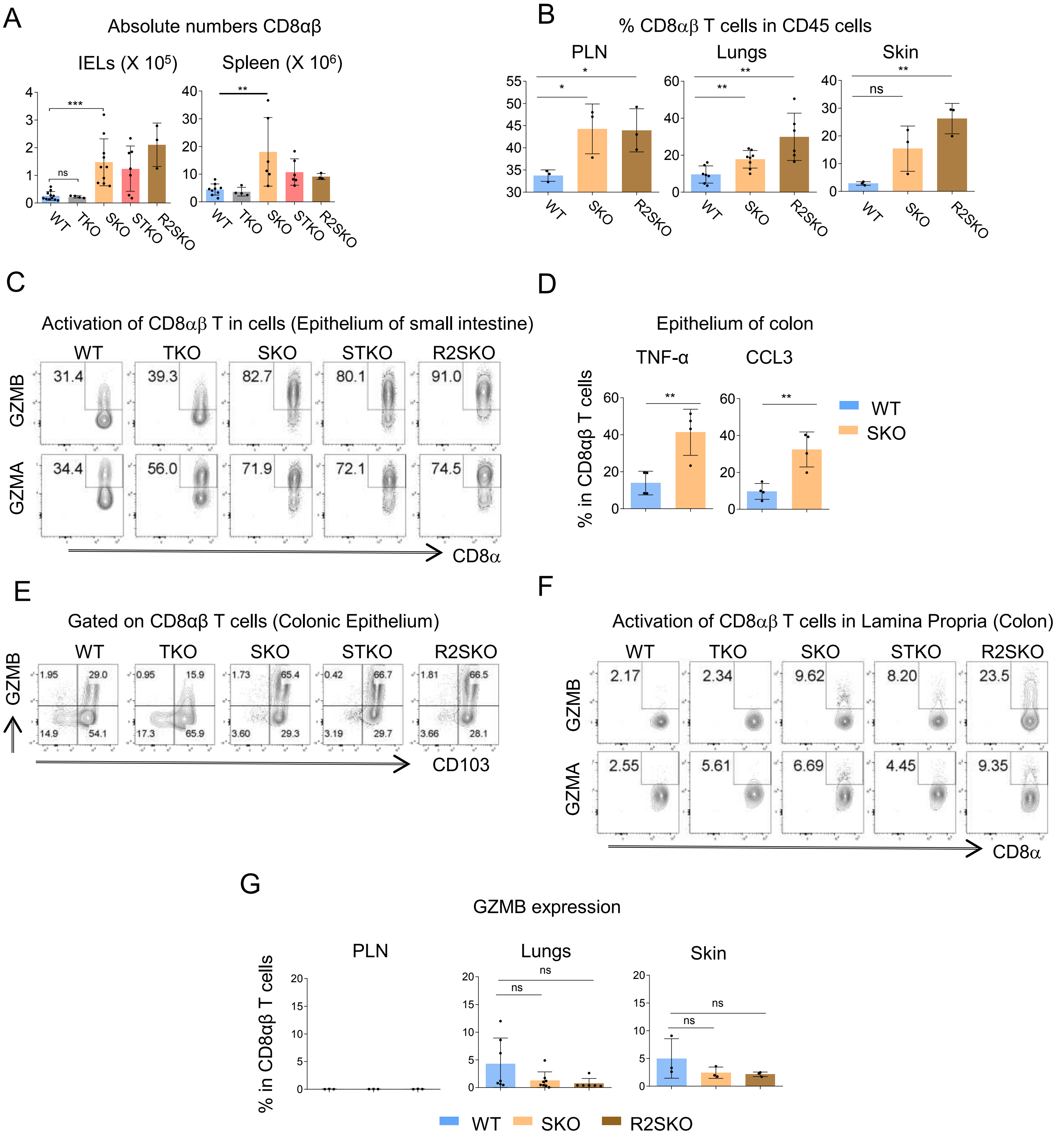
A
Absolute numbers CD8αβ
IELs (X 10^5)
Spleen (X 10^6)
WT TKO SKO STKO R2SKO
B
% CD8αβ T cells in CD45 cells
PLN
Lungs
Skin
WT SKO R2SKO
C
Activation of CD8αβ T in cells (Epithelium of small intestine)
WT TKO SKO STKO R2SKO
GZMB
31.4 39.3 82.7 80.1 91.0
GZMA
34.4 56.0 71.9 72.1 74.5
CD8α
D
Epithelium of colon
TNF-α
CCL3
% in CD8αβ T cells
WT
SKO
E
Gated on CD8αβ T cells (Colonic Epithelium)
WT TKO SKO STKO R2SKO
GZMB
CD103
F
Activation of CD8αβ T cells in Lamina Propria (Colon)
WT TKO SKO STKO R2SKO
GZMB
2.17 2.34 9.62 8.20 23.5
GZMA
2.55 5.61 6.69 4.45 9.35
CD8α
G
GZMB expression
PLN
Lungs
Skin
% in CD8αβ T cells
WT SKO R2SKO

Supplemental Figure 7

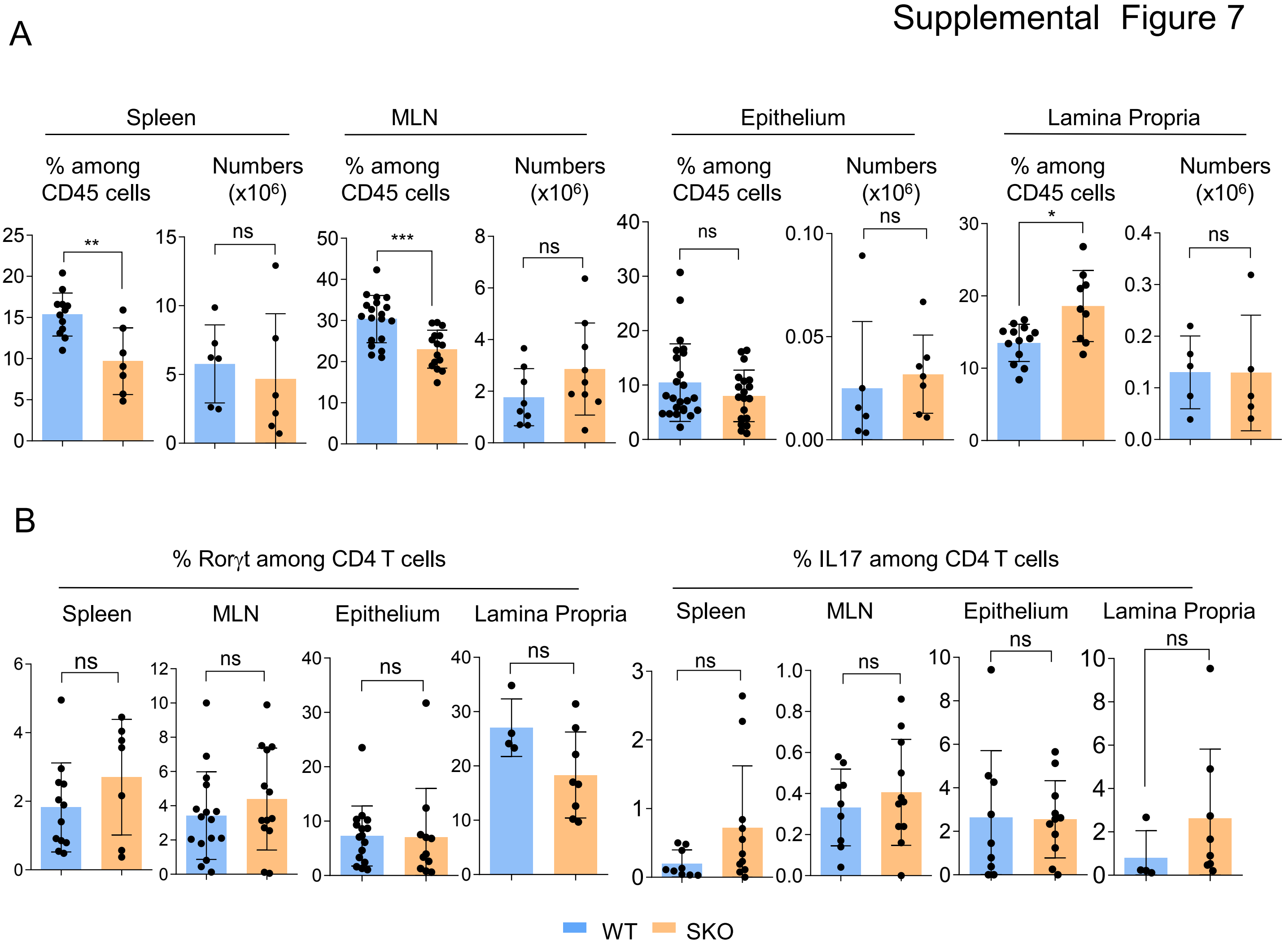

Supplemental Figure 8

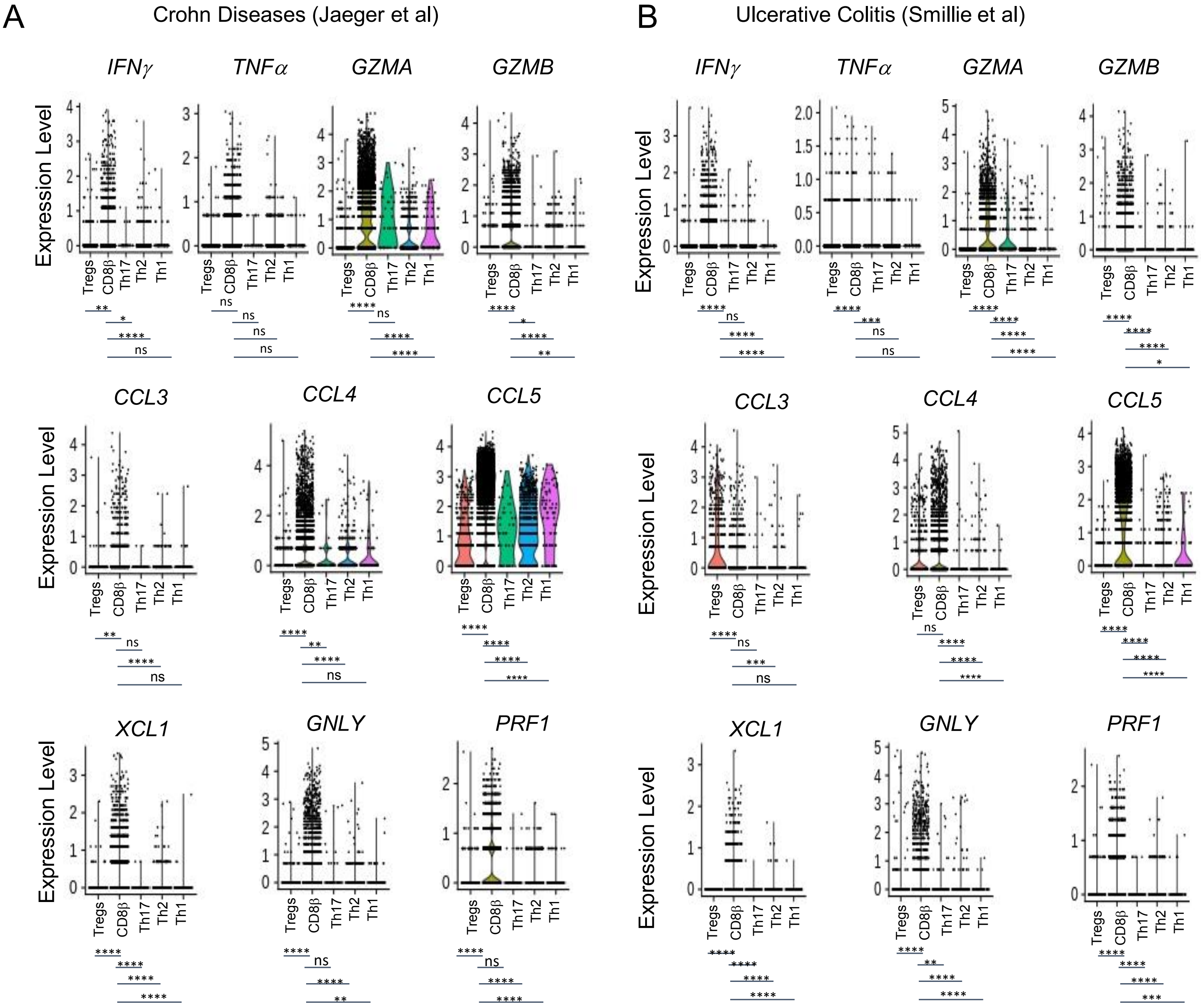

Supplemental Figure 9

A

F5 transgenic mice RAG2KO

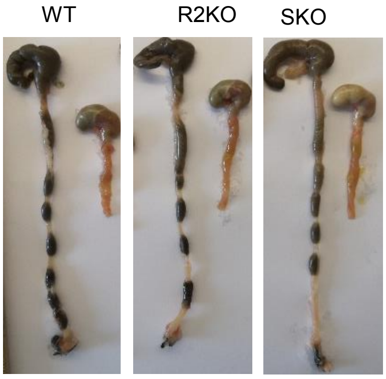

B

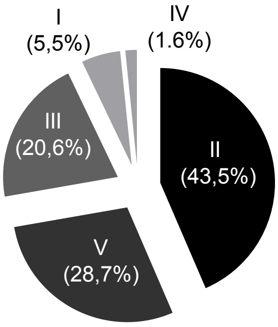

C

Gene Set Enrichment Analysis (GSEA)

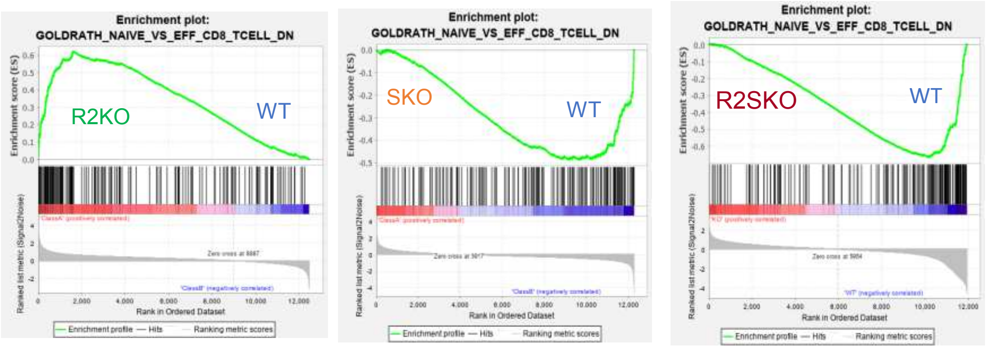

D

List of genes used for violin plot

Ccl3 Ccl4 Ccl5 Ccr2 Ccr4 Ccr5 Cd244a Cxcr3 Fasl Gzma Gzmb Id2 Ikzf2 Il12rb1 Il12rb2 Il2ra Il2rb Il2rg Irf4 Irf8 Itgax Klra2 Klra7 Klrb1b Klrb1f Klrc1 Klri2 Klrk1 Prdm1 Prf1 Rora Tbx21 Xcl1 Zeb2 Mki67 Ctla4 Cdk1 Cd44 Bhlhe40 Itga1 Il18rap Pde4a Eomes

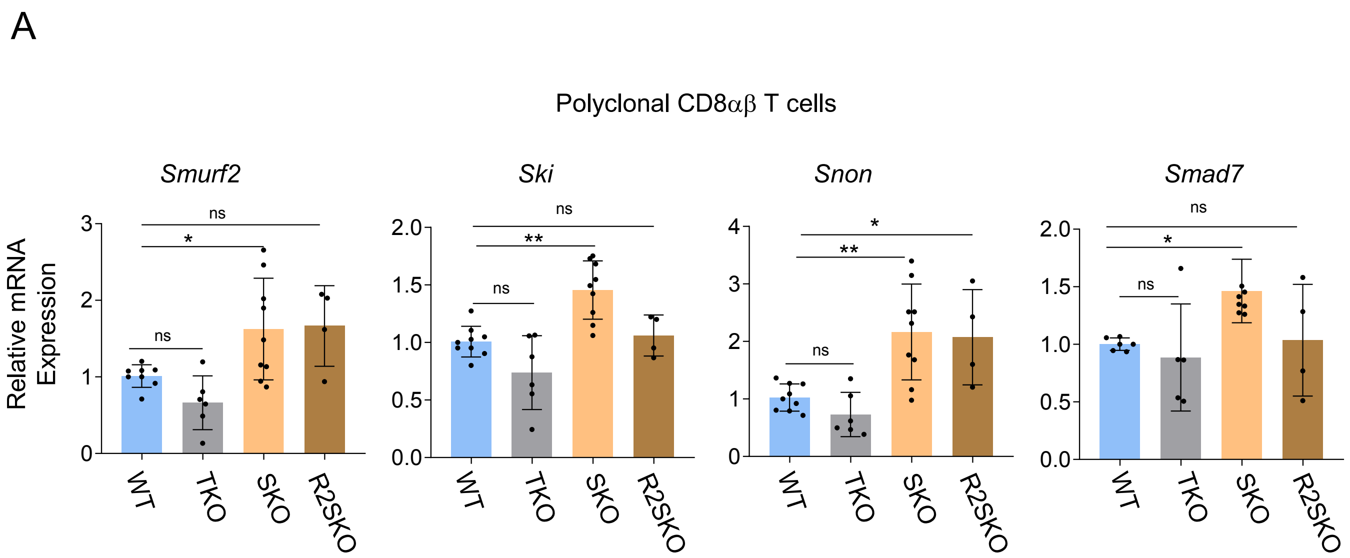
A
Polyclonal CD8αβ T cells
Smurf2
Ski
Snon
Smad7
Relative mRNA Expression
ns
*
**
WT
TKO
SKO
R2SKO

Supplemental Figure 11

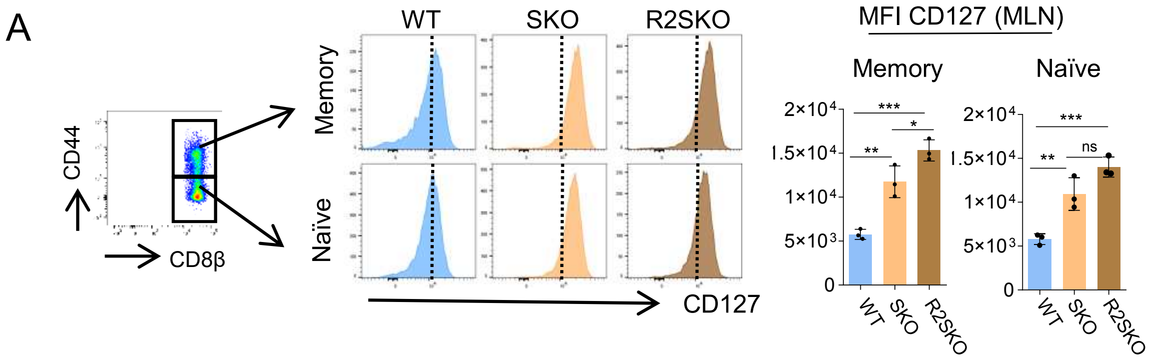

Supplemental Figure 12

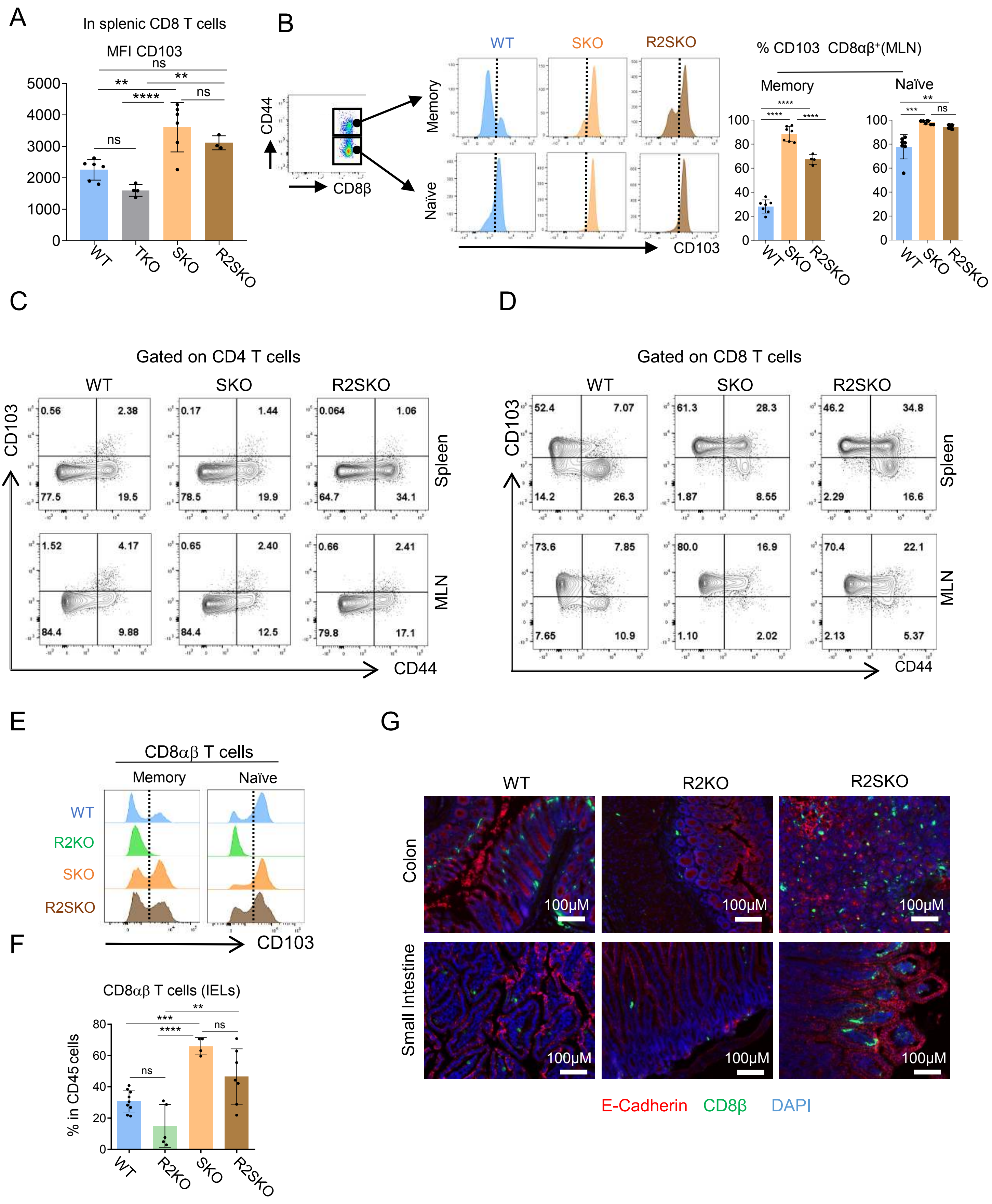

Supplemental Figure 13

A

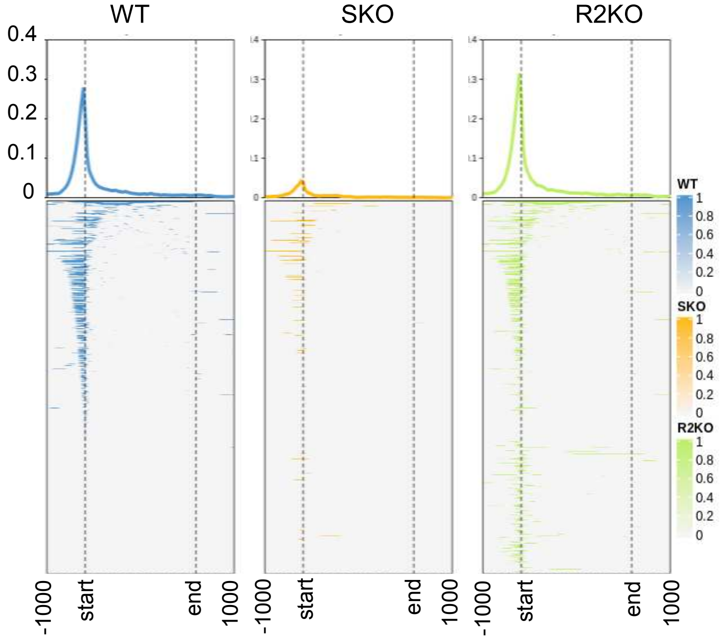

C

List of the 103 genes

Sgk1 Ly6e Kcnc1 Sfr1 Arrb2 Atp8b4 F2r Tnk2 Fam3c Il12rb1 Ly6c2 Cxcr6 Ift80 Prkcz Ccr9 Snord110 Golm1 Nt5e Arhgef12 Tgfb1i1 Smurf2 Smpd5 Pde3b P2rx7 St8sia6 Pdlim1 Ski Baiap3 Ifngr1 Gm8369 Ly6i Rgs10 Celsr1 Smad3 Il6ra Slc6a19 Rasal1 Fmnl3 Enc1 Lpxn Snord32a Vmp1 Snord34 Slc16a5 Atxn1 Snord49b Btg1 Zfp605 Rab3ip Dzip1 Fyn Gimap7 Camsap2 Kctd12 Dnajb1 Cd163l1 Dtx1 Myh9 Dennd4a Ttc28 Susd3 Xdh Snord57 Lrig1 Relb Ly6a Eef2k Fam102a Skil Rora Tcf4 Slfn5 Itgae Podnl1 Lef1 Actb Wnt5b Tfrc Dad1 1700017B05Rik Smad7 Gng2 Erdr1 Arl4c Tnfrsf14 Cobll1 Irf6 Oasl2 Coro2a Tmem64 Tle3 Abi3 Cd3d Pde4a Marc1 Lpin2 Spsb1 Gpr68 Emid1 Tgfbr2 E2f2 Cotl1 Gdpd

B

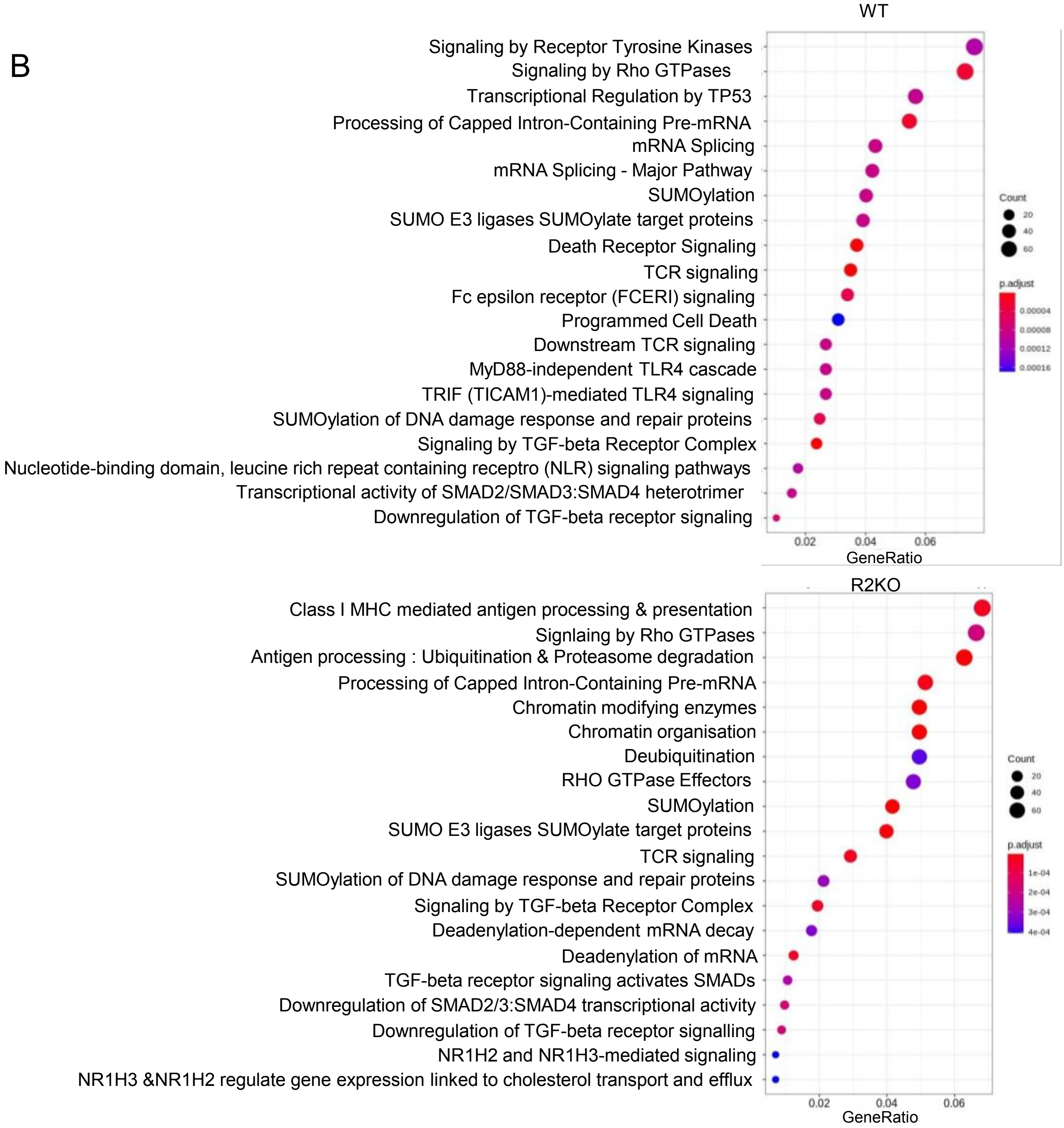

Supplemental Figure 14

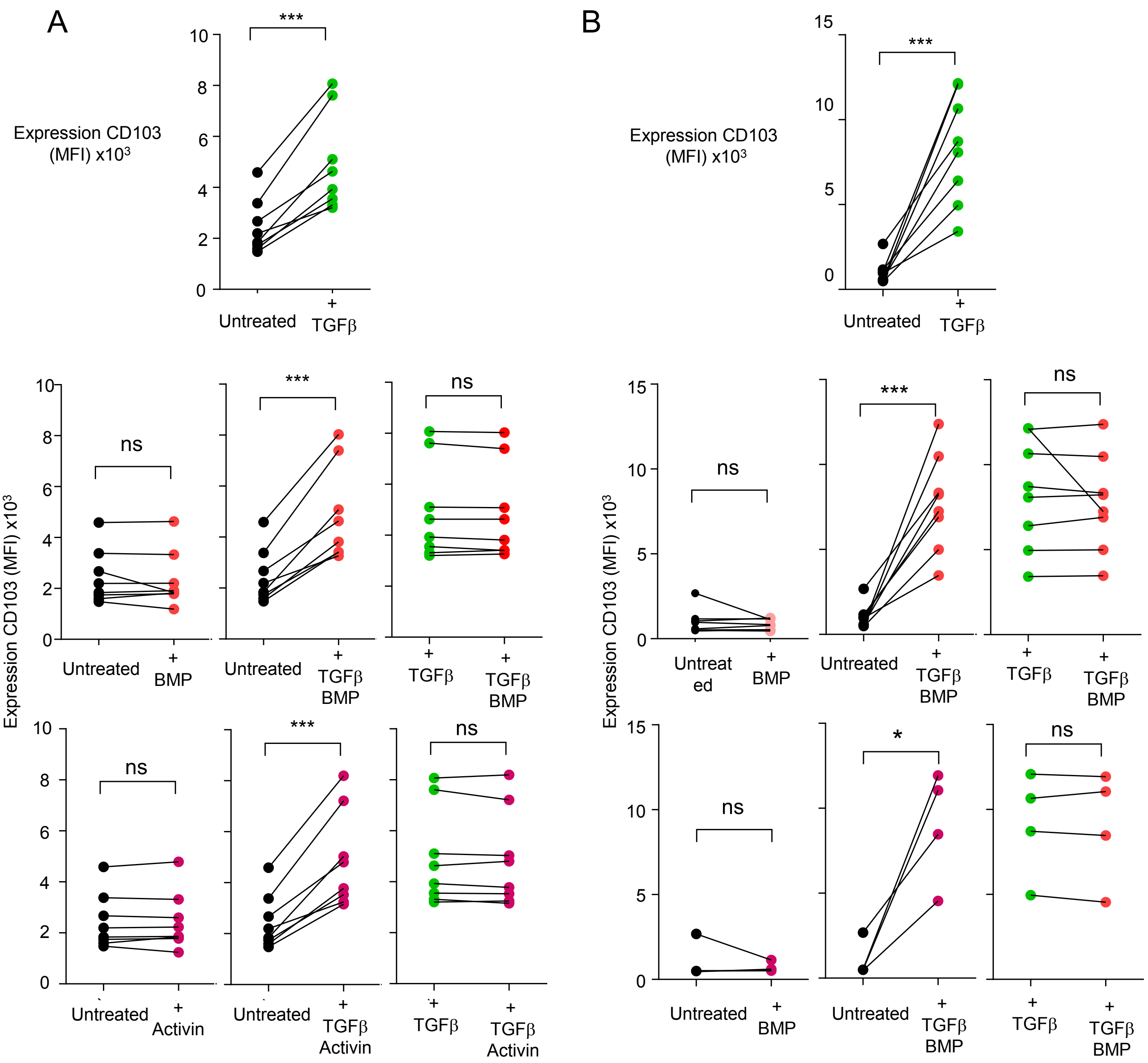

Supplemental figure legends

Supplemental Figure 1 (relative to Figure 1). (A): Flow cytometry staining of SMAD4 and TGFβ-RII in T cells and non-T cells from WT, TKO, SKO, STKO and R2SKO mice**.** (representative of 2 experiments, n=6) **(B):** Representative H&E staining of duodenum and colon sections from 7 months-aged WT, TKO, SKO, STKO and R2SKO mice. Scale bar represents 100µm. Original magnification, ×20. All data represent at least 2 independent experiments, n = 3 mice minimum.

Supplemental Figure 2 (relative to Figure 1). (A): Representative H&E staining of duodenum and colon sections of irradiated RAG2KO mice reconstituted with WT, R2KO, SKO, or R2SKO BM cells. Scale bar represents 100µm. Original magnification, ×20.

Supplemental Figure 3 (relative to Figure 2). (A-B): Representative flow cytometry plots showing the frequency of CD4 and CD8αβ T cells after CD8β or CD4 antibodies treatment **(A)**; TCRγδ and CD8αα **(B)** among CD45 cells in the MLN and intra epithelial lymphocytes of irradiated RAG2KO mice reconstituted with WT BM cells and injected with PBS or anti-CD8β or anti-CD4 depleting antibody. **(C):** Frequencies and numbers of CD8αβ, TCRγδ and CD8αα T cells in the IEL from the colon and small intestine of irradiated RAG2KO mice reconstituted with WT or SKO BM cells and injected with PBS or anti-CD8β. All data represent at least 3 independent experiments and are presented as mean ± SD. Each symbol represents an individual mouse, n = 3 mice minimum. Data were analyzed by unpaired Student t test. ns: nonsignificant; * $p<0,05$; ** $p < 0.01$; *** $p < 0.001$; **** $p < 0.0001$.

Supplemental Figure 4 (relative to Figure 2). (A): Change of body weight at day 35-40 after SKO (n=5-7 per condition) or WT (n=3-6 per condition) BM reconstitution and treatment with

IgG isotypes (rat IgG1 isotype and rat IgG2b Isotype) or anti-CD8β or anti-CD4 antibodies. **(B):** Representative H&E staining of duodenum and colon sections of irradiated RAG2KO mice reconstituted with WT or SKO BM cells and treated with anti-CD4 or anti-CD8β depleting antibodies. Scale bar represents 200µm. Original magnification, ×20. **(C):** Representative pictures of colon and body weight shown as relative to WT of irradiated RAG2KO mice reconstituted with WT or R2SKO BM cells and treated with anti-CD8β depleting antibody or PBS. Data are presented as mean ± SD. Each symbol represents an individual mouse. This data is representative of 2 experiments. Data were analyzed by one-way ANOVA followed by Tukey's multiple comparison test for other panels. ns: nonsignificant; * p<0,05; **p < 0.01; ***p < 0.001; ****p < 0.0001.

Supplemental Figure 5 (relative to Figure 2). (A-C): Experimental procedure for mixed BM chimera generation. RAG2KO mice were irradiated and reconstituted with 50/50 mixed WT and R2SKO BM cells. Plots show cells gated on total $CD4^{+}$ T cells revealing the presence of both WT (CD45.1) and R2SKO (CD45.2) CD4 T cells **(A)**; Flow cytometry plots showing the presence of CD4 Tregs **(B)**; Photo of the colon (**C**); Change of body weight (**C)** at 35 days post-reconstitution **(C)**. All data represent at least 2 independent experiments

Supplemental Figure 6 (relative to Figure 3). (A): Histograms showing the absolute numbers of CD8αβ T cells in the spleen and colonic intra epithelial lymphocytes of 7 months aged WT, TKO, SKO, STKO and R2SKO mice. **(B):** Histograms showing the frequency of CD8αβ T cells in the peripheral lymph nodes (PLN), lungs and the skin of 7 months-aged WT, SKO and R2SKO mice. **(C):** Flow cytometry staining of GZMA and GZMB in CD8αβ T cells of the epithelium of the small intestine of WT, TKO, SKO, STKO and R2SKO mice. **(D):** Histograms showing the frequency of TNF-α and CCL3 producing CD8αβ T cells in the colonic epithelium

of 7 months-aged WT and SKO mice. **(E):** CD103 and GZMB staining showing that activated CD8αβ T cells are mainly CD103 positive and are present in the intestinal epithelium. **(F):** Flow cytometry staining of GZMA and GZMB in the colonic lamina propria CD8αβ T cells of WT, TKO, SKO, STKO and R2SKO mice. **(G):** The frequency of GZMB producing CD8αβ T cells in the PLN, lungs and the skin of 7 months-aged WT, SKO and R2SKO mice. All data represent at least 3 independent experiments and are presented as mean ± SD. Each symbol represents an individual mouse. n = 3 or more for each group. Data (A, B, and G) were analyzed by one-way ANOVA followed by Tukey's multiple comparison test. Data (panel D) were analyzed by unpaired Student t test. ns: nonsignificant; * p<0,05; **p < 0.01; ***p < 0.001; ****p < 0.0001.

Supplemental Figure 7 (relative to Figure 3). (A): Frequency and numbers of CD4 T cells in the spleen, MLN, IEL, and Lamina Propria of colon from WT or SKO mice aged between 5 and 8 months. **(B):** Frequency of Rorγt and IL17 expressing CD4 T cells in the spleen, MLN, IEL, and Lamina Propria from WT or SKO mice aged between 5 and 8 months. All data represent at least 3 independent experiments and are presented as mean ± SD. Each symbol represents an individual mouse (n = 4 or more for each group). Data were analyzed by unpaired Student t test. ns: nonsignificant; * p<0,05; **p < 0.01; ***p < 0.001; ****p < 0.0001.

Supplemental Figure 8 (relative to Figure 3). (A-B): Analysis of effector gene expression in human intestinal T cells from Jaeger's single cell RNA-seq data and Smillie single cell RNAseq data as reported in Methods. The Violin plots show the distribution of indicated effector molecule expression (in arbitrary units) cross each cell type in CD **(A)** or UC **(B)** patients. P value for pairwise comparisons between groups is shown in the plot. * P<0.05, ** P<0.01, *** P<0.001, ****P<0.0001 (two-tailed Student's t test).

Supplemental Figure 9 (relative to Figure 4). (A): Representative pictures of colon and duodenum from F5 TCR transgenic WT, R2KO and SKO 8 months-aged mice. (n=3-4 mice per group) **(B):** Pie chart showing the frequency of each cluster (related to Figure 4B). **(C):** Gene Set Enrichment Analysis (GSEA) plot comparing gene expression arrays related to naïve or effector state of WT (n=4), R2KO (n=3), SKO (n=3), and R2SKO (n=4) CD8αβ T cells. **(D):** List of the 43 selected genes related to the CD8 T cell effector state.

Supplemental Figure 10 (relative to Figure 5). (A): Quantitative RT-PCR analysis of the expression of indicated TGF-β regulatory genes in polyclonal CD8αβ T cells from spleen of WT, TKO, SKO, and R2SKO mice. All data represent at least 3 independent experiments and are presented as mean ± SD. Each symbol represents an individual mouse. Data were analyzed by one-way ANOVA followed by Dunnett's multiple comparison test (All were compared to WT). ns: nonsignificant; * $p<0,05$; ** $p < 0.01$; *** $p < 0.001$; **** $p < 0.0001$.

Supplemental Figure 11 (relative to Figure 7). (A): Flow cytometry staining of CD127 expression on naïve (CD44 negative CD8 T cells) and memory (CD44 positive CD8 T cells) in the MLN of WT, SKO and R2SKO mice. All data represent at least 3 independent experiments and are presented as mean ± SD. Each symbol represents an individual mouse. Data were analyzed by one-way ANOVA followed by Tukey's multiple comparison test. ns: nonsignificant; * $p<0,05$; ** $p < 0.01$; *** $p < 0.001$; **** $p < 0.0001$.

Supplemental Figure 12 (relative to Figure 8). (A): Histograms showing the expression of CD103 in splenic CD8 T cells from WT, TKO, SKO, and R2SKO mice. **(B):** Flow cytometry staining of CD103 on naïve (CD44 negative) or memory (CD44 positive) CD8 T cells from the MLN of WT, SKO and R2SKO mice. **(C-D):** Flow cytometry staining of CD103 versus CD44

expression on CD4 T cells **(C)** compared to CD8 T cells from the MLN and spleen of WT, SKO and R2SKO mice **(D). (E):** CD103 expression on naïve (CD44-) and memory ($CD44^{pos}$) CD8 T cells from irradiated and transplanted mice. **(F)** Histogram showing the frequency of CD8αβ T cells among CD45 cells in the colonic epithelium of RAG2KO irradiated mice and reconstituted with WT, R2KO, SKO or R2SKO BM cells **(G):** Representative pictures showing immune-fluorescence staining of CD8β (green), E-cadherin (red), DAPI (blue) in the small intestine and colon sections of irradiated RAG2KO mice reconstituted with WT, R2KO, or R2SKO BM cells. All data represent at least 3 independent experiments and are presented as mean ± SD. Each symbol represents an individual mouse. Each symbol represents an individual mouse, n = 4 mice minimum. Data were analyzed by one-way ANOVA followed by Tukey's multiple comparison test. ns: nonsignificant; * p<0,05; **p < 0.01; ***p < 0.001; ****p < 0.0001.

Supplemental Figure 13 (relative to Figure 10). (A): Enriched heatmaps showing the SMAD4-occupancy signals in WT, SKO and R2KO (SKO was used as a negative control). **(B):** Biological pathway enrichment analysis in SMAD4 binding genes in WT and R2KO CD8 T cells. **(C):** List of common genes that have a SMAD4 positive binding peak and are deferentially expressed between WT, SKO and R2KO naïve F5 CD8αβ T cells.

Supplemental Figure 14 (relative to Figure 10). (A-B): Naïve F5 transgenic CD8 T cells were cultured with or without 10 ng/ml of TGF-β1 or 10 ng/ml of BMP2 and/or 10 ng/ml of Activin. 3 days later, the expression of CD103 was determined on CD8 T cells. **(A):** Cells were stimulated by the different indicated cytokines. **(B):** Cells were stimulated with anti CD3/CD28 conjointly to the different indicated cytokines. Data represent a pool of 3 independent experiments and are presented as mean ± SD. Each symbol represents an individual mouse, n=8

per group. Data were analyzed by paired t test. ns: non-significant; * $p<0,05$; ** $p < 0.01$; *** $p < 0.001$; **** $p < 0.0001$.
